# Hematopoietic Antigen Presenting Cells Fine‐Tune the Effector Phenotype of Intrathymic Unconventional αβ T Cells

**DOI:** 10.1002/eji.70243

**Published:** 2026-07-22

**Authors:** Yi Wang, Trang T. Lam, Sotaro Ochiai, Abbie R. Larson, Dean B. Matthews, Hamish E.G. McWilliam, Tsuneyasu Kaisho, Mireille H. Lahoud, Jose A. Villadangos, Gretchen E. Diehl, Franca Ronchese, Daniel G. Pellicci, Mark M.W. Chong

**Affiliations:** ^1^ St Vincent's Institute of Medical Research Fitzroy Australia; ^2^ Department of Medicine (St Vincent's) University of Melbourne Fitzroy Australia; ^3^ Malaghan Institute of Medical Research Wellington New Zealand; ^4^ Immunology Program of the Sloan Kettering Institute Memorial Sloan Kettering Cancer Center New York New York USA; ^5^ Immunology and Microbial Pathogenesis Program Weill Cornell Graduate School of Medical Sciences New York New York USA; ^6^ Department of Microbiology and Immunology, Peter Doherty Institute for Infection and Immunity University of Melbourne Parkville Australia; ^7^ Department of Biochemistry and Pharmacology Bio21 Molecular Science and Biotechnology Institute University of Melbourne Parkville Australia; ^8^ Industry‐Government‐Academia Collaboration Promotion Headquarters Wakayama Medical University Kimiidera Japan; ^9^ Department of Biochemistry Monash Biomedicine Discovery Institute Monash University Clayton Australia; ^10^ Murdoch Children's Research Institute Parkville Australia; ^11^ Department of Paediatrics University of Melbourne Parkville Australia

## Abstract

The development of unconventional αβ T cells, including invariant natural killer T (iNKT) and mucosal‐associated invariant T (MAIT) cells, in the thymus is distinct from conventional T cells. Unconventional αβ T cells adopt a memory phenotype, acquire effector functions, and can reside in the thymus long‐term. It is well‐established that positive selection of these unconventional T cells from CD4^+^CD8^+^ double‐positive (DP) precursors depends on interaction with other DP cells. However, postselection, the regulation of their maturation and effector differentiation is less well understood. Professional antigen‐presenting cells (APCs) are thought to have a role, but their roles have only been partially investigated previously. In this study, we investigate the impact of perturbing thymic dendritic cell (DC) and macrophage populations on intrathymic iNKT and MAIT effector subsets in C57BL/6 mice. We show that conventional type 1 DCs (cDC1s) support iNKT1 cells, while CX3CR1‐ and Mgl2‐expressing cDC2s and macrophages support MAIT17 cells. Lastly, we show that disrupting the XCR1‐XCL1 axis, which was previously shown to control cDC1 localization to the thymic cortex, alters the balance between iNKT and MAIT cells in the thymus, where there is augmentation of the iNKT cell compartment at the expense of MAIT cells. These findings further highlight the roles of hematopoietic APCs in supporting intrathymic unconventional αβ T cells.

## Graphical abstract

Our study describes how the effector maturation of intrathymic iNKT and MAIT cells is guided by distinct hematopoietic APCs: DC1s for iNKT cells and macrophages for MAIT cells.

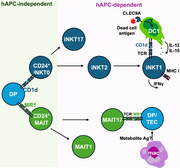

## 1 Introduction

MAIT cells and iNKT cells are unconventional T cells that, like other αβ T cells, develop from CD4^+^CD8^+^ thymocyte precursors in the thymus [1]. Commitment to these lineages depends on positive selection through recognition of their cognate antigen‐presenting molecules on other CD4^+^CD8^+^ thymocytes, CD1d for iNKT cells, and MR1 for MAIT cells. Subsequently, both lineages upregulate PLZF, a master regulator of the unconventional T cell program, and undergo further maturation by downregulating CD24 and upregulating CD44 [2]. Subsets of naïve CD44^−^ iNKT cells and MAIT cells then exit the thymus, becoming the precursors of the peripheral iNKT and MAIT cell pools [3]. The cells that remain in the thymus continue to differentiate, adopting a memory phenotype and acquiring effector function. In C57BL/6 mice, thymic MAIT cells differentiate into IL‐17‐producing RORγt^+^ MAIT17 cells or IFN‐γ‐producing Tbet^+^ MAIT1 cells, while thymic iNKT cells mostly differentiate into iNKT1 cells, with a small proportion differentiating into iNKT17 [4, 5]. An additional PLZF^hi^ iNKT2 population has also been identified. Of these, those that express IL‐4 are terminally differentiated iNKT2 cells, while the other IL‐4 nonproducers are thought to be precursors of thymic iNKT1 cells [5]. This dominance of specific effector phenotypes varies between mouse strains [6].

The extrinsic signals that guide these changes and lineage diversification are only partly understood. Thymic epithelial cells (TEC) are important for iNKT differentiation via cytokines like IL‐15 and IL‐25 that promote iNKT1 and iNKT2, respectively [7]. In return, RANKL from immature iNKT cells supports TEC differentiation [8]. IL‐4 production by iNKT2 cells requires activation by macrophages [9]; the IL‐4 from iNKT2 cells regulates intrathymic cDC1 differentiation [10]. For the MAIT lineage, the differentiation of intrathymic MAIT17 cells requires microbial‐derived RibD‐dependent metabolite antigens produced by bacteria, but how these are transported to the thymus and which APCs are responsible are unknown [11]. Complicating the assessment of which APCs are different in effector phenotypes between mouse strains, and the finding that thymic DCs and macrophages are far more heterogeneous than previously thought [12, 13, 14].

Using various genetic mouse models to deplete DCs and macrophages, we investigate how these APCs influence the effector phenotypes of intrathymic iNKT and MAIT cells in C57BL/6 mice. We show that cDC1s support iNKT1 cells, whereas Mgl2 or CX3CR1‐expressing cDC2s and/or macrophages support MAIT17 cells. Additionally, we show how mislocalization of cDC1s alters the balance between iNKT and MAIT cells in the thymus. Our findings contribute to a growing appreciation of the importance of hematopoietic APCs for the maintenance of intrathymic unconventional αβ T cells.

## 2 Results

### 2.1 Thymic Hematopoietic APCs Are Predicted to Interact With Developing iNKT and MAIT Cells

**FIGURE 1:**
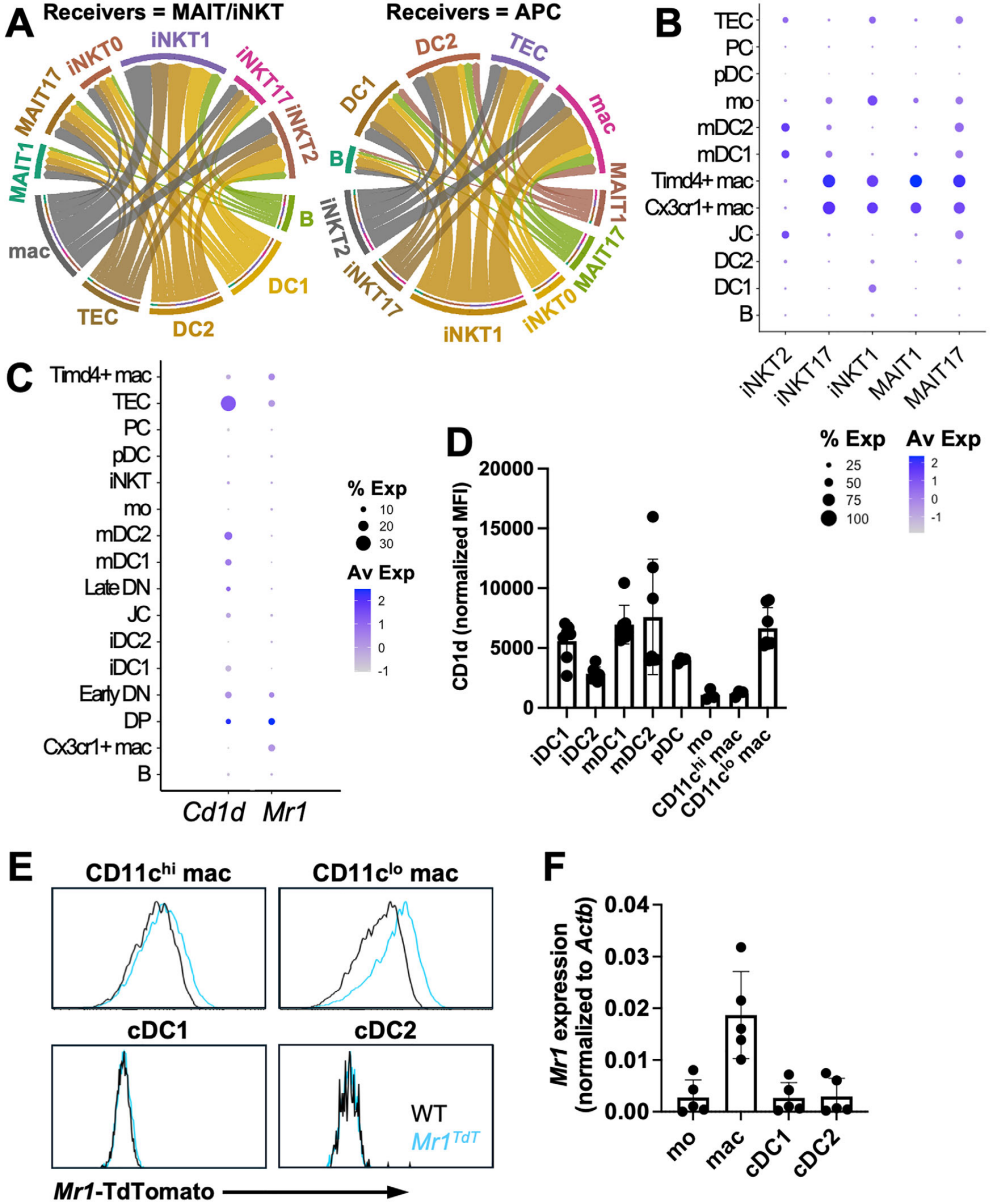
Thymic myeloid APCs are predicted to interact with MAIT and iNKT cells. (A) Frequency cord diagrams predicting the interactions between mouse thymic APCs and MAIT/iNKT subsets based on gene expression profiles extracted from scRNA‐seq data of cells from C57BL/6 mice. Shown is the analysis of iNKT or MAIT cells as receivers (left) or APCs as receivers (right). Extracted gene lists are provided in Table S1. (B) Dotplot of predicted interaction scores between iNKT and MAIT subsets and APC populations. (C) Dotplot of *Cd1d1* and *Mr1* expression by thymic populations from mouse scRNA‐seq. (D) The indicated populations in the thymus of 6–7‐week‐old C57BL/6 mice were analyzed for CD1d surface expression by FACS. Each dot represents an individual animal analyzed (*n* = 6), performed over three experiments. The gating strategy for identifying thymic APC populations is shown in Figure S2. (E) The thymus of *Mr1^TdTomato^* and wildtype mice were analyzed for TdTomato expression by FACS as a reporter for *Mr1* gene transcription. Shown are representative histograms of the TdTomato signal in thymic CD11c^hi^ macrophages (which are also CX3CR1^+^), CD11c^lo^ macrophages (which are also TIMD4^+^), cDC1 and cDC2 cells. (F) The indicated populations were sorted from the thymus of 6–7‐week‐old C57BL/6 mice and analyzed for *Mr1* mRNA expression by qRT‐PCR. Each dot represents an individual sort (*n* = 5).

To further investigate if hematopoietic APCs might influence intrathymic unconventional T cells, we started with in silico interaction analyses by integrating previously generated single‐cell RNA sequencing (scRNA‐seq) datasets of thymic APCs [13], iNKT cells [5], and MAIT cells [15] from C57BL/6 mice. The clusters were annotated (Figure S1A–E) according to these previous publications, then interaction predictions were conducted with the R package LIANA [16]. Along with TECs, thymic DCs and macrophages were predicted to mediate a large proportion of the interactions with MAIT and iNKT cells, with little contribution from B cells (Figure 1A). We also extracted genes differentially expressed by MAIT or iNKT subsets and selected those encoding secreted or cell surface proteins (Figure S1F; Table S1), to predict if there is preferential interaction with either cDC1s, cDC2, macrophages, B cells, or TECs. This predicted that macrophages have the highest interactions with iNKT17, MAIT1 and MAIT17, cDC1s with iNKT1, and mature DCs (both mDC1 and mDC2) with iNKT2 cells (Figure 1B).

Next, we assessed the expression of CD1d, which presents antigen to iNKT cells. Based on the scRNA‐seq data, cDC1s, mature DCs, and some macrophages express *Cd1d* RNA, which was comparable to DP thymocytes (Figure 1C). We also confirmed CD1d protein expression by FACS (Figure S2A; Figure 1D).

As for expression of MR1, which presents antigen to MAIT cells, both the CD11c^lo^ (which are also TIMD4^+^) and CD11c^hi^ (which are also CX3CR1^+^Mgl2^+^) macrophages [13], express *Mr1* RNA at levels comparable to DP thymocytes and TECs (Figure 1C). Steady state surface MR1 is difficult to detect, but is upregulated upon stimulation with the MR1 ligand 5‐OP‐RU [17]. Thymic CX3CR1^+^ cDC2s (Figure S2B,C) and CD11c^hi^ macrophages (Figure S2D,E) mildly upregulated MR1 following stimulation. We also assessed the *Mr1^TdTomato^* knock‐in reporter mouse [18] without the need for stimulation, which confirmed expression by thymic macrophages (Figure 1E). This was further confirmed by RT‐PCR for *Mr1* RNA (Figure 1F). This cell‐type‐specific expression of CD1d or MR1 could indicate that different thymic APCs may have roles in supporting either intrathymic iNKT or MAIT cells.

### 2.2 Thymic Hematopoietic APCs Physically Interact With Developing iNKT and MAIT Cells

To determine if thymic APCs can interact with thymic unconventional T cells, we exploited thymic rosettes, which are cellular complexes of a single APC interacting with a ring of thymocytes. We previously showed these are enriched for hematopoietic APCs and thymocytes postpositive selection [13]. Previous high‐throughput flow imaging also suggested enrichment of TCR‐MHC at APC‐thymocyte interfaces [13], suggesting that these rosettes are capturing a snapshot of APCs interacting with late‐stage maturing thymocytes.

**FIGURE 2:**
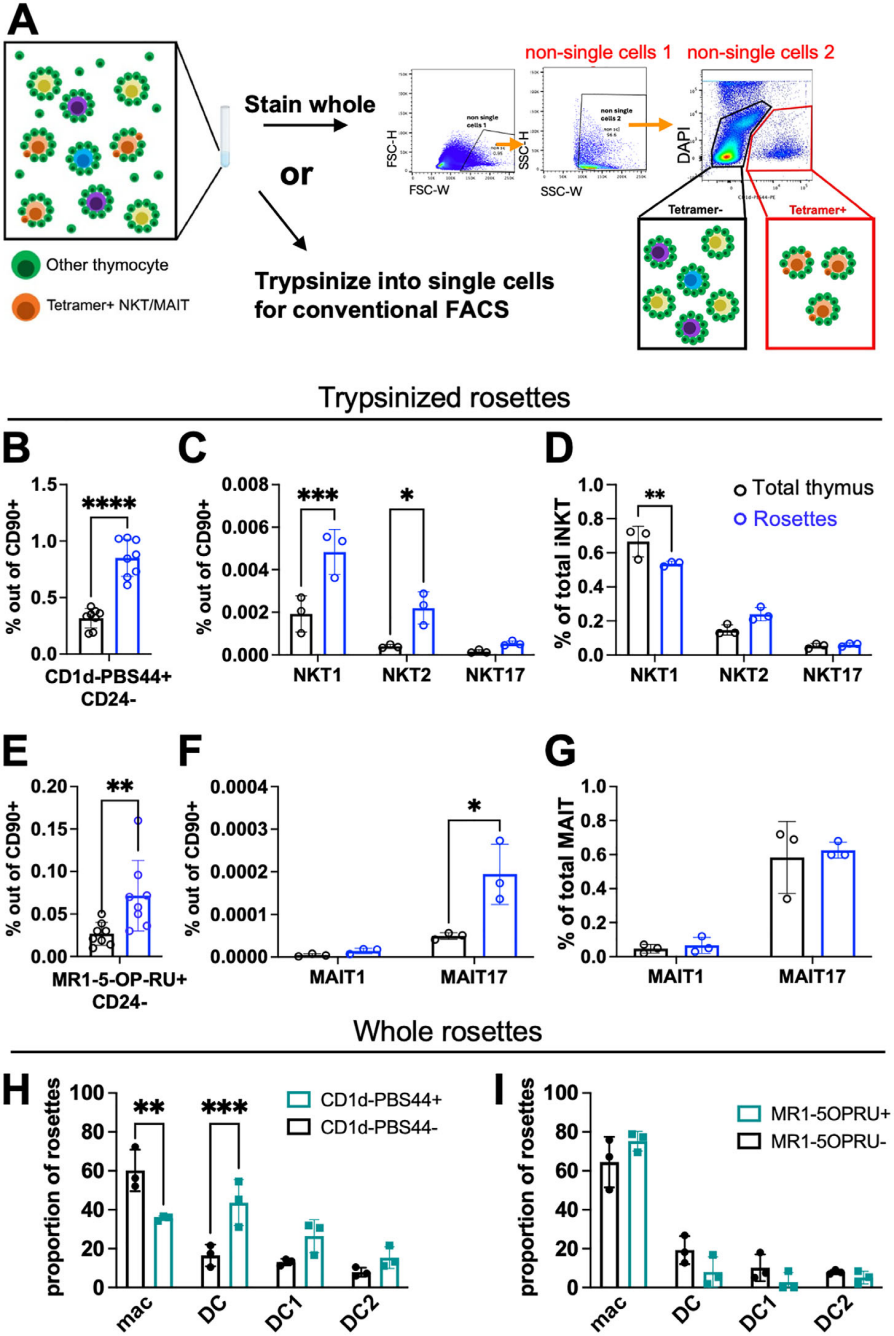
Thymic myeloid APCs physically interact with developing iNKT and MAIT cells. (A) Experimental setup for analyzing physical interactions between APCs and iNKT or MAIT cells in thymic rosettes. Rosettes were isolated from the thymus of 6–7‐week‐old C57BL/6 mice. These were either trypsinized into single cells for conventional FACS or analyzed as whole rosettes to identify specific APCs found within either iNKT or MAIT cell‐containing rosettes. First, isolated rosettes were trypsinized into single cells and compared with single cell suspensions of total thymus for (B) mature iNKT cells (identified as CD1d‐PBS44^+^TCRβ^+^CD24^−^ cells) (*n* = 8), (C) iNKT effector subsets based on transcription factor expression out of total thymocytes (*n* = 3), and (D) effector subsets out of total iNKT cells (*n* = 3). The data shows the percentage of the indicated populations out of total CD90^+^ thymocytes. Single‐cell suspensions of rosettes were compared with single‐cell suspensions of total thymus for (E) mature MAIT cells (identified as MR1‐5‐OP‐RU^+^TCRβ^+^PLZF^+^CD24^−^ cells) (*n* = 8), (F) MAIT effector subsets based on transcription factor expression out of total thymocytes (*n* = 3), and (G) effector subsets out of total MAIT cells (*n* = 3). The gating strategy for identifying thymic iNKT and MAIT populations is shown in Figure S3. When analyzing for MAIT cells, the cells were first blocked with MR1‐6FP. FACS analysis of whole rosettes stained for (H) iNKT or (I) MAIT cells along with antibodies for APC markers. Rosettes were first blocked with an Fc receptor binding inhibitor polyclonal antibody before antibody/tetramer staining. Shown are the percentages of tetramer^+^ or tetramer^−^ rosettes that contain the indicated macrophage or DC population. For all graphs, each dot represents an independent experiment, where the thymuses of three mice were pooled. *p < 0.05, **p < 0.01, ***p < 0.005, ****p < 0.001 (two‐way ANOVA with Šídák's multiple comparisons test or unpaired *T*‐test).

We isolated rosettes from C57BL/6 mice and investigated if there might be enrichment of iNKT or MAIT cells (Figure 2A). If there is increased iNKT or MAIT identification within rosettes, it would suggest that these cells are interacting with APCs in these clusters. Unconventional T cells were identified by FACS with tetramers. While CD24^+^ immature iNKT and MAIT cells are present in the thymus, these are very rare and difficult to quantify by FACS [19]. We therefore focused only on mature CD1d‐PBS44^+^TCRβ^+^CD24^−^ iNKT thymocytes and mature MR1‐5‐OP‐RU^+^TCRβ^+^PLZF^+^ CD24^−^ MAIT thymocytes (Figure S3A,B). These were then subdivided into effector subsets based on expression of the lineage‐defining transcription factors Tbet, RORγt, and PLZF (Figure S3C,D). Analysis of dissociated rosettes found that both iNKT and MAIT cells (Figure 2B–G) are enriched in thymic rosettes, particularly the T‐bet^+^ iNKT1 and RORγt^+^ MAIT17 subsets, which are the dominant effector subsets in the C57BL/6 mice.

We also analyzed whole rosettes by FACS to determine which APCs might be interacting with thymic iNKT and MAITs. Whole rosettes were first stained with the MR1‐5‐OP‐RU or CD1d‐PBS44 tetramers, then interrogated for enrichment of specific APCs in tetramer^+^ rosettes compared with tetramer^−^ rosettes (Figure S3E). There was an enrichment of DCs and concurrent depletion of macrophages in CD1d‐PBS44‐labeled rosettes (Figure 2H), and no enrichment of any specific APC in MR1‐5OP‐RU‐labelled rosettes, though there was a trend of reduced DCs (Figure 2I).

### 2.3 A Reduction in Intrathymic iNKT Cells Without cDC1s

**FIGURE 3:**
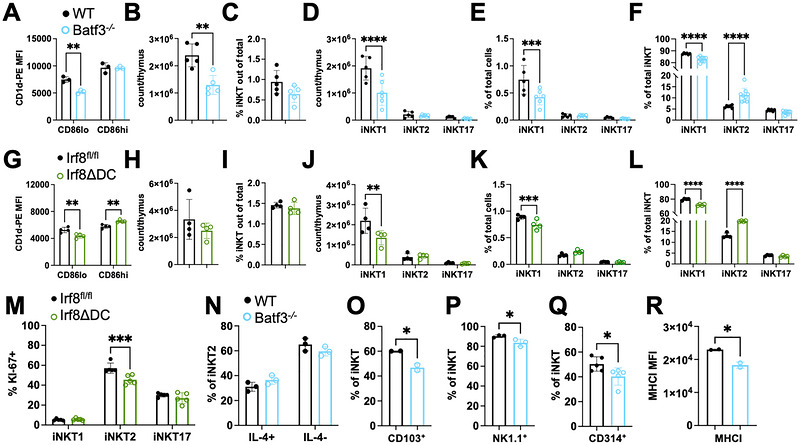
Intrathymic iNKT1 cells are reduced in C57BL/6 mice that lack cDC1s. (A) Comparison of surface CD1d expression on immature (CD86^lo^) and mature (CD86^hi^) F4/80^−^CD11c^+^MHCII^+^ DCs in the thymus of 6–7‐week‐old *Batf3^−/−^* and littermate WT mice (*n* = 3). The thymus of *Batf3^−/−^* mice were analyzed by FACS for (B) the number of mature iNKT cells per thymus, (C) % iNKT out of total thymocytes, (D) the number of each iNKT effector subset per thymus, (E) iNKT effector subset out of total thymocytes, and (F) effector subset out of total iNKT cells. (G) Comparison of surface CD1d expression on DCs in the thymus of *Irf8^fl/fl^ CD11c‐cre* (Irf8ΔDC) and littermate *Irf8^fl/fl^* control mice. The thymus of Irf8ΔDC mice were analyzed by FACS for (H) the number of mature iNKT cells per thymus, (I) % iNKT out of total thymocytes, (J) the number of each iNKT effector subset per thymus, (K) iNKT effector subset out of total thymocytes, and (L) effector subset out of total iNKT cells. (M) FACS analysis for Ki‐67^+^ proliferating cells within each iNKT effector subset in Irf8ΔDC mice. (N) FACS analysis for the frequency of IL‐4^+^ and IL‐4^−^ cells with the PLZF^+^Tbet^−^RORγt^−^ iNKT2 population in *Batf3^−/−^* mice. FACS analysis for the frequency of (O) CD103^+^, (P) NK1.1^+^, and (Q) NKG2D^+^ cells, and (R) MHCI mean fluorescence intensity in the thymic iNKT1 subset of *Batf3^−/−^* mice. For all graphs, each dot represents an individual animal analyzed, where each experiment involved the analysis of *Batf3^−/−^* and WT littermate pair or Irf8ΔDC and *Irf8^fl/fl^* and littermate pair. *p < 0.05, **p < 0.01, ***p < 0.005, ****p < 0.001 (Two‐way ANOVA with Šídák's multiple comparisons test or unpaired *T*‐test).

To investigate how DCs influence intrathymic iNKT cells, we analyzed the phenotype of mouse models with perturbations in DC populations, all of which were on the C57BL/6 background. We started with *Batf3^−/−^* mice commonly employed as a model of cDC1 deficiency [20]. In the thymus, BATF3 expression is enriched in cDC1s and is not expressed by thymocytes (Figure S4), and we previously confirmed that thymic cDC1s are absent in *Batf3^−/−^* mice [13]. In addition, CD86^lo^ immature DCs had reduced CD1d expression (Figure 3A; Figure S5A). In these mice, there was a reduction in iNKT cells (Figure 3B,C) that primarily affected the T‐bet^+^ iNKT1 subset (Figure 3D–F; Figure S5B). Expression of IFNγ or IL‐4 by these effector subsets was also confirmed by intracellular staining following in vitro re‐stimulation (Figure S5C). We also repeated these iNKT subset analyses by first enriching for CD1d‐PBS44^+^ thymocytes with MACS magnetic bead selection. The same reduction in iNKT1 cells was observed (Figure S5D–E).

Although BATF3 is best known as a master regulator of the cDC1 lineage, increasing evidence suggests that it may have functions in other cell types, potentially within certain T cells [21, 22]. We therefore also analyzed Irf8ΔDC mice in which the *Irf8* gene is deleted by CD11c‐cre. While *Irf8* is expressed by multiple cell types (Figure S4), CD11c‐cre mediated deletion results in cDC1 deficiency in the thymus, and there is also a 50% reduction in thymic macrophages [13]. Like *Batf3^−/−^* mice, the remaining thymic cDCs in these mice displayed a reduction in CD1d expression by the CD86^lo^ immature cells, but increased CD1d expression by CD86^hi^ mature cells (Figure 3G; Figure S5A). Although not significant, there was a trend of reduced iNKT cells overall (Figure 3H,I), and like *Batf3^−/−^* mice, Irf8ΔDC mice showed a significant reduction in iNKT1 cells (Figure 3J–L; Figure S5F). The identical phenotype of *Batf3^−/−^* and Irf8ΔDC mice thus suggests that cDC1s have a role in supporting intrathymic iNKT1 cells.

The reduction in iNKT1 cells does not appear to be due to decreased proliferation, indicated by Ki‐67 staining, though there was a reduction in the frequency of Ki‐67^+^ iNKT2 cells (Figure 3M).

Intrathymic iNKT2 cells are comprised of two subsets. Those that produce IL‐4 are terminally differentiated iNKT2 cells, while those that do not produce IL‐4 are thought to be the precursors of iNKT1s [5]. We did not observe any change in the proportion of IL‐4^+^ to IL‐4^−^ iNKT2 cells in In *Batf3^−/−^* mice, suggesting that the loss of NKT1s is probably not due to defects in their precursors (Figure 3L; Figure S5C).

CD103, NK1.1, and CD314 (or NKG2D) are that are upregulated (Figure S5G) as iNKT cells mature from iNKT0 to iNKT1 cells [23, 24, 25]. We additionally identified MHCI as another marker (Figure S5H). In *Batf3^−/−^* mice, the expression of all these markers by thymic iNKT1 cells were mildly lower (Figure 3O–R), which is consistent with the reduction in iNKT1 cells observed.

### 2.4 The Phenotype of iNKT1 Cells Is Supported by Cdc1s

**FIGURE 4:**
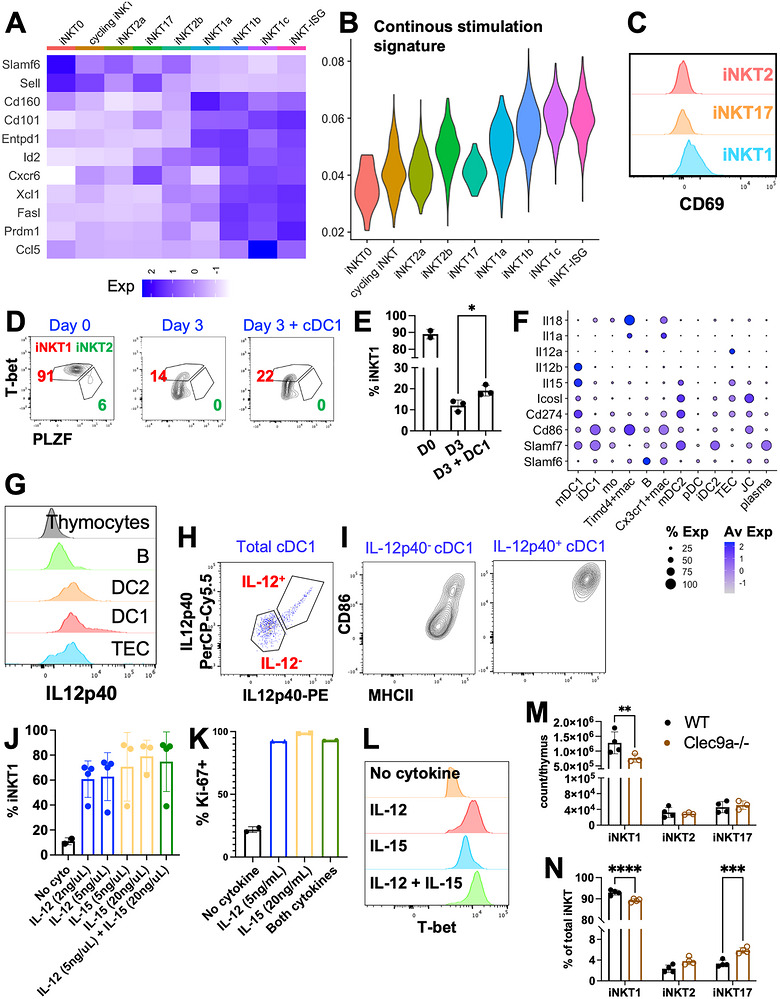
cDC1s stabilize the phenotype of thymic iNKT1s via cytokines and dead cell antigen processing. (A) Heatmap of pseudobulk expression of genes associated with continuous CD8^+^ T cell stimulation, obtained from Wherry et al. [26], in thymic iNKT subpopulations identified in the scRNA‐seq dataset generated by Baranek et al. [1]. The gene set is shown in Table S2. (B) Modulescore for the expression of this stimulation gene set in the indicated thymic iNKT subpopulations. (C) FACS analysis for CD69 expression on thymic iNKT subsets in 6–7‐week‐old C57BL/6 mice. (D) CD8‐depleted thymocytes from C57BL/6 mice were cultured in vitro in the presence or absence of sorted thymic cDC1s. After 3 days, the CD1d‐PBS44^+^TCRβ^+^CD24^−^ iNKT cells were analyzed for T‐bet and PLZF expression by FACS. A representative FACS is shown. (E) Pooled data from multiple culture experiments. Each dot represents an independent experiment. (F) Dotplots of genes expressed by thymic APCs that are predicted to mediate interactions with MAIT and iNKT cells (from the scRNA‐seq analysis of Figure 1). (G) Representative FACS analysis for IL12p40 protein expression by thymic APCs. (H) Representative FACS analysis for IL12p40 expression in total thymic cDC1s. (I) IL‐12p40^+^ and IL‐12p40^−^ cDC1s were analyzed for maturation status. (J) CD8‐depleted thymocytes were cultured for 3 days with the indicated cytokines, then the frequency of T‐bet^+^ iNKT1 cells out of total iNKT cells was quantified by FACS. Each dot represents an independent experiment. (K) FACS analysis for Ki‐67 in iNKT1 cells at the end of the 3‐day culture. (L) Representative FACS analysis for T‐bet expression in iNKT1 cells at the end of the 3‐day culture. The thymus of 7–8‐week‐old *Clec9a^−/−^* and littermate WT mice were analyzed by FACS for (M) the number of each iNKT effector subset per thymus and (N) effector subset out of total iNKT cells. Each dot is of an individual animal analyzed, where each experiment involved the analysis of a *Clec9a^−/−^* and 1 or 2 WT littermate pair. **p < 0.01, ***p < 0.005, ****p < 0.001 (two‐way ANOVA with Šídák's multiple comparisons test).

As thymic iNKT cells differentiate toward the iNKT1 subset, we noticed the acquisition of a gene expression profile that is very similar to the signature in CD8^+^ T cells that receive continuous stimulation [26]. The downregulation of *Slamf6* and upregulation of *Entpd1*, *Prdm1*, *Cxcr6*, and *Xcl1* (Figure 4A,B; Table S2), in particular, are associated with this phenotype, typically termed “exhaustion” [27]. Though unlikely to be actual T cell exhaustion, this phenotype in thymic iNKT1 cells could be related to their long‐term retention in the thymus and extended interactions with cDC1 cells. Consistent with this, iNKT1 cells upregulate CD69 [28, 29]. We confirmed that thymic iNKT1 cells indeed expressed higher CD69 than iNKT2 or iNKT17 cells in C57BL/6 mice (Figure 4C).

To further investigate how cDC1s might influence thymic iNKT1 cells, we turned to in vitro culture of CD8‐depleted thymocytes from C57BL6 mice, which was previously shown to enrich for thymic iNKT cells [7]. We found that after the 3d culture, most iNKT1 cells had lost T‐bet expression, but this was partially rescued by the addition of sorted thymic cDC1s (Figure 4D,E).

Trans‐presentation of IL‐15 by TECs stimulates thymic iNKT1 cell proliferation [7], while IL‐12 is well‐known to drive Th1 immunity [30]. Analysis of the thymic APC scRNA‐seq dataset suggests that cDC1s highly express *Il15* and *Il12b* (encoding IL‐12p40) RNA (Figure 4F). At the protein level, both cDC1s and cDC2s, as well as TECs, in the thymus of C57BL/6 mice express IL‐12p40, but a subset of cDC1s appeared to express particularly high levels (Figure 4G), which were found to be CD86^hi^MHCII^hi^ mature cells (Figure 4H,I).

To further investigate the function of IL‐12 and IL‐15 on thymic iNKT cells, we again employed the CD8‐depleted thymocyte cultures. As previously reported [7], IL‐15 resulted in a greater frequency of iNKT1 cells in these cultures that was associated with increased proliferation (Figure 4J,K). IL‐12 also induced a comparable expansion. We also found that these cytokines, especially IL‐12, strongly upregulated T‐bet expression (Figure 4L). Thus, cDC1s may be stabilizing the phenotype of thymic iNKT1 cells via cytokines like IL‐12 and IL‐15 that can stimulate iNKT1 cell proliferation and induce T‐bet expression.

Finally, we investigated whether the antigen processing machinery of cDC1 cells may have a role by analyzing *Clec9a* ^−/−^ mice. CLEC9A is uniquely expressed by cDC1s (Figure S4), where it binds to dead cell debris and facilitates phagosome rupture [31]. While the CLEC9A pathway promotes cross‐presentation of dead cell peptide antigens via MHCI, intracellular trafficking of dead cell debris could also deliver the lipid antigen pool to CD1d. Thymic DC populations, at least by broad lineage, appeared to be normal in *Clec9a* ^−/−^ mice (Figure S6A), as was CD1d expression by these cells (Figure S6B). However, we observed a reduction in iNKT cells (Figure 4M,N; Figure S6C) that was similar in *Batf3^−/−^* and Irf8ΔDC mice, suggesting a role for cDC1s in supporting intrathymic iNKT1 cells.

### 2.5 cDC2s and Macrophages Are Not Essential for Intrathymic iNKT1 Cells

As mature cDC2s and macrophages in the thymus also express CD1d (Figure 1C,D), we next investigated if these cells might support intrathymic iNKT cells in C57BL/6 mice. Macrophages were previously shown to be important for the maturation of iNKT2 cells in the thymus of BALB/c mice [9].

We previously showed that thymic cDC2s, which are distinct from their counterparts found in peripheral lymphoid organs, have a highly overlapping transcriptional signature with thymic macrophages [13]. Both thymic cDC2s and CD11c^hi^ macrophages express CX3CR1 and CD301b/Mgl2 (Figure S4). We started by employing CX3CR1‐DTR mice [32] to deplete these populations. After 10 days of DT treatment, we confirmed that cDC2s are entirely depleted from the thymus, along with half the macrophages that correspond to the CD11c^hi^ subset that also expresses CX3CR1 (Figure S7A,B). However, we found no discernible impacts on thymic iNKT cells (Figure S8C–G). DT‐treatment of Mgl2‐DTR mice, which depletes thymic cDC2s and the same CD11c^hi^ macrophage subset [13], also had no impact on thymic iNKT cells (Figure S7H–L). Thus, compared with cDC1s and cDC2s, macrophages appear to be less important for supporting intrathymic iNKT cells in C57BL/6 mice.

### 2.6 Thymic Cdc2/Macrophage Depletion Skews the Intrathymic MAIT Effector Subset Balance

We next analyzed the various DC/macrophage models for intrathymic MAIT cells. We first excluded a role for cDC1s because thymic MAIT cells were largely normal in *Batf3^−/−^* mice (Figure S9A–E). MACS‐enrichment of MR1‐5‐OP‐RU^+^ thymocytes before FACS analysis also does not reveal any changes in MAIT subsets in *Batf3^−/−^* mice (Figure S9F,G).

**FIGURE 5:**
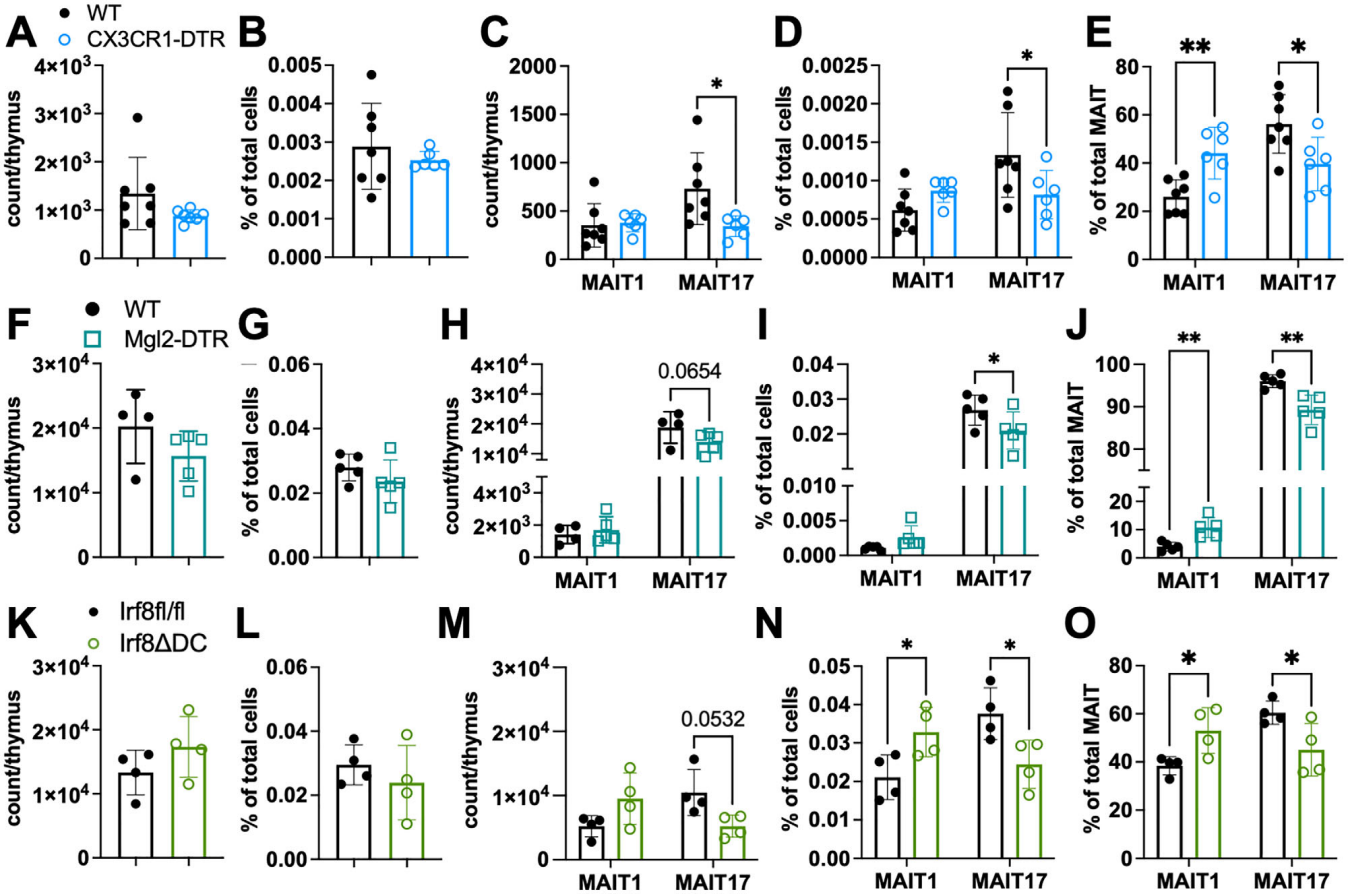
Intrathymic MAIT7 cells are partially dependent on cDC2s or macrophages. 6–8‐week‐old CX3CR1‐DTR and littermate WT mice were DT‐treated for 10 days, then the thymus was analyzed by FACS for (A) the number of mature MAIT cells per thymus, (B) % MAIT cells out of total thymocytes, (C) the number of each MAIT effector subset per thymus, (D) MAIT effector subset out of total thymocytes, and (E) effector subset out of total MAIT cells. 6‐week‐old Mgl2‐DTR and littermate WT mice were DT‐treated for 10 days, then the thymus was analyzed by FACS for (F) the number of mature MAIT cells per thymus, (G) % MAIT cells out of total thymocytes, (H) the number of each MAIT effector subset per thymus, (I) MAIT effector subset out of total thymocytes, and (J) effector subset out of total MAIT cells. The thymus of 6–7‐week‐old Irf8ΔDC mice and littermate controls were analyzed by FACS for (K) the number of mature MAIT cells per thymus, (L) % MAIT cells out of total thymocytes, (M) the number of each MAIT effector subset per thymus, (N) MAIT effector subset out of total thymocytes, and (O) effector subset out of total MAIT cells. For all graphs, each dot represents an individual animal analyzed, where each experiment involved the analysis of a DTR and WT littermate pair, or Irf8ΔDC and *Irf8^fl/fl^* littermate pair. *p < 0.05, **p < 0.001 (two‐way ANOVA with Šídák's multiple comparisons test).

When we examined the CX3CR1‐DTR model, we found no impact on overall MAIT cells (Figure 5A,B), but there was a significant skewing away from the MAIT17 effector phenotype (Figure 5C–E; Figure S9A). Analysis of Mg2‐DTR mice found the same skewing away from the MAIT17 effector phenotype without affecting overall MAIT cell frequencies (Figure 5F–J; Figure S9B). We noted differences in total MAIT cell numbers between the wildtype littermates of the CX3CR1‐DTR and Mgl2‐DTR colonies, most likely because of differences in microbial exposure in the different breeding locations. Notwithstanding, the identical phenotype in both DTR models suggests that thymic cDC2s and/or macrophages play a role in supporting intrathymic MAIT17 cells.

Next, we analyzed Irf8ΔDC mice, which we previously showed to have a reduction in thymic macrophages (in addition to cDC1 deficiency) [13]. We again observed the skewing away from the MAIT17 effector phenotype without changes in overall MAIT cell frequencies (Figure 5K–O; Figure S9C). Because *Batf3^−/−^* mice did not exhibit a thymic MAIT phenotype, this suggests that it is the depletion of the macrophages that is likely affecting intrathymic MAIT cells in Irf8ΔDC mice.

Finally, because macrophages have the highest expression of MR1 in the thymus (Figure 1C,E,F), we wanted to investigate whether this MR1 is important. For this, we analyzed *Mr1* ^fl/fl^ *Lyz2^cre^* (Mr1cKO) mice that lack MR1 expression specifically in myeloid cells [18]. We confirmed that surface MR1 expression by thymic macrophages, at least the low levels that can be detected, is also lost (Figure S10A). However, we did not observe any impact on thymic MAIT cells in these mice (Figure S10B–G).

### 2.7 Disruption of the XCL1–XCR1 Axis Alters Both MAIT and NKT Cell Development

The immediate precursors of iNKT and MAIT cells are thought to share the same developmental niche in the thymus due to their common requirement of interacting with other DP thymocytes for positive selection [1, 33]. Thus far, the changes in MAIT and iNKT effector phenotypes we observed were due to the loss of either cDC1s or cDC2/macrophages, and the changes in one unconventional T cell lineage were independent of the other. This suggests that after positive selection, thymic iNKT and MAIT cells may no longer be competing for the same developmental niche.

**FIGURE 6:**
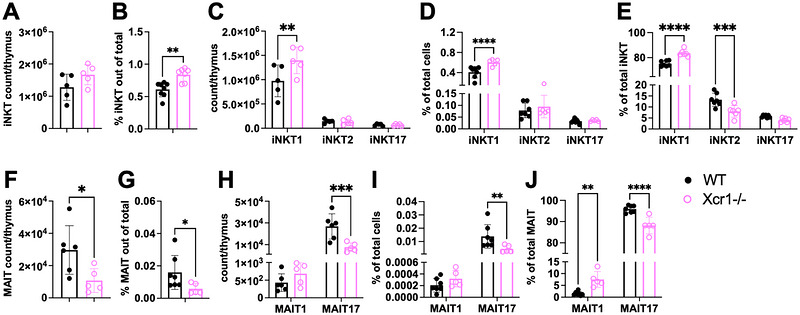
Disruption of the XCL1‐XCR1 axis alters the balance between intrathymic iNKT and MAIT cells. The thymus of 6‐week‐old *Xcr1^−^* ^/−^ and littermate WT mice were analyzed by FACS for (A) the number of mature iNKT cells per thymus, (B) % iNKT cells out of total thymocytes, (C) the number of each iNKT effector subset per thymus, (D) iNKT effector subset out of total thymocytes, and (E) effector subset out of total iNKT cells. The mice were also analyzed by FACS for (F) the number of mature MAIT cells per thymus, (G) % MAIT cells out of total thymocytes, (H) the number of each MAIT effector subset per thymus, (I) MAIT effector subset out of total thymocytes, and (J) effector subset out of total MAIT cells. For all graphs, each dot represents an individual animal analyzed, where each experiment involved the analysis of an *Xcr1^−^* ^/−^ and a WT littermate. *p < 0.05, **p < 0.01, ***p < 0.005, ****p < 0.001 (two‐way ANOVA with Šídák's multiple comparisons test).

XCL1 by medullary TEC mediates the medullary‐localization of cDC1s, which express XCR1, and ablating this chemokine axis results in the mislocalization of cDC1s to the cortex [34]. While XCR1 deficiency did not affect overall cDC1 numbers (Figure S11A) or CD1d expression by DCs (Figure S11B) in the thymus, we observed a significant increase in intrathymic iNKT cells, notably iNKT1 cells (Figure 6A–E; Figure S11C,D). In contrast, we observed a reduction in intrathymic MAIT cells, particularly MAIT17 cells (Figure 6I,J; Figure S11E,F). This reciprocal impact on intrathymic iNKT and MAIT cells resembles the previously described niche‐disruption phenomenon [33]. This phenotype of *Xcr1^−/−^* mice therefore suggests that there are differences in the developmental niches of immature and mature stages (Figure S12).

Together with the demonstration of APC‐specific impacts on iNKT and MAIT effector phenotypes, these findings reveal important new insights into how hematopoietic APCs interact with and support the intrathymic unconventional T cells in C57BL/6 mice.

## 3 Discussion

It is clear that the selection of unconventional T cells from DP thymocyte precursors is dependent on interactions with other CD1d and MR1‐expressing DP thymocytes. However, the effector differentiation and maintenance of intrathymic unconventional T cells continues to be clarified. There is a high degree of variation in the effector phenotypes between mouse strains [6], which can complicate the interpretation of phenotypes caused by individual genetic perturbations. Potentially, their maturation and effector diversification could simply be stochastic, but it is thought that there are guiding cues because broad perturbations in thymic stromal/APC populations affect these later stages. For example, transgenic expression of CD1d by hematopoietic cells but not TECs is effective at mediating the negative selection of iNKT cells [35], while RelB deficiency in TECs impairs the maturation of iNKT cells in thymic grafts [36].

While mTECs have previously been shown to regulate iNKT1 maturation [7, 8], in this study, we showed that cDC1s also participate in the supporting intrathymic iNKT1 maturation, possibly by providing cytokines and antigen stimulation. Interestingly, we found that *Clec9a* ^−/−^ mice exhibited the same iNKT1 phenotype as cDC1‐deficient mice. CLEC9A is uniquely expressed by cDC1s and is required for cross‐presentation of peptide antigens. This finding suggests that this pathway may also be facilitating presentation to maturing iNKT cells, presumably lipid antigens. Though speculation, lipid antigens from dead cell debris could be loaded onto CD1d as a byproduct of the rupture of phagosomes mediated by CLEC9A/DNGR‐1 [31]. Clearly, this potential connection needs to be directly addressed in the future, but our data suggest that in addition to the cytokines produced, the specialized antigen processing machinery of cDC1s may also have a role in supporting the homeostasis of intrathymic iNKT1 cells.

It was previously shown in BALB/c mice that CD1d expression by CD11c^+^ macrophages is important for iNKT2 cell numbers in the thymus and for their ability to secrete IL‐4, while neither iNKT1 nor iNKT17 cells were dependent on macrophages [9]. On the surface, this appears to be in contrast to our observation that intrathymic iNKT1 cells are affected by cDC1s but not macrophages or cDC2s depletion. However, in BALB/c mice, iNKT cells are predominantly of the iNKT2 effector phenotype, while in C57BL/6 mice, iNKT1 cells dominate [6]. It is also important to note that our DTR models, although highly effective at depleting cDC2s, were only partially effective at depleting macrophages. This was sufficient to affect thymic MAIT17 maturation but may not be sufficient to affect iNKT2 maturation. Moreover, we previously showed that Irf8ΔDC mice, which lack cDC1s, are partially depleted of thymic macrophages [13], and in this study, we showed that these mice also exhibited a similar MAIT17 phenotype as the CX3CR1‐DTR and Mgl2‐DTR models.

Specifically assessing the in vivo functions of cDC2s or macrophages remains a challenge, especially in the thymus, due to their complex ontogeny and their overlapping phenotype [37]. The genetic models we used to assess their functions in the thymus disrupted both lineages, while the population that has been defined as cDC2s is, in fact, comprised of multiple lineages [12, 13]. That being said, both cDC2s and adult monocyte‐derived macrophages are migratory APCs, and we showed that they have considerable transcriptional overlap between them in the thymus, particularly in humans [13]. This could be a mechanism that provides for functional redundancy between these populations in the event that one lineage is lost.

In addition to the difference between mouse strains, there are important differences in unconventional T cells between mouse and human. In humans, the intrathymic unconventional T cells often exhibited a mixed phenotype, expressing both T‐bet and RORγt [38, 39, 40]. These cells can produce IFN‐γ but only low levels of IL‐17A [38, 41]. Moreover, MAIT cells are more dominant in humans, whereas iNKT cells are more dominant in mice [42]. Given these differences, it will be important to understand if there is a similar support of unconventional T cell development provided by thymic hematopoietic APCs in the human thymus.

The lack of a MAIT phenotype in macrophage‐specific MR1cKO mice suggests that antigen representation via MR1 by these cells is dispensable, either because antigen is not required or because there is redundant presentation by another cell type. Paiva et al. [43] recently showed that CD11c‐cre‐mediated deletion of *Mr1*, which should delete in both thymic DCs and macrophages, also does not affect MAIT cell development. CX3CR1^+^ transendothelial or transitional DCs enter the thymus at the corticomedullary junction [12, 13, 44], and other cells in the cortex, such as DP thymocytes, could potentially have access to the microbial antigens they carry. Even nonphagocytes like thymocytes and TECs can present 5‐OP‐RU [11] and thus redundancy in the provision of metabolite antigens to developing MAIT cells in the thymus is quite possible. Indeed, it was shown that both thymocyte and TEC‐specific MR1 deficiency impairs thymic MAIT development, but neither completely disrupts development [43].

In most of the genetic models of perturbed hematopoietic APC populations that we analyzed, there were either impacts only on thymic iNKT cells or only on MAIT cells, suggesting that the two lineages may be regulated independently, possibly due to distinct spatial localization. The exception was in *Xcr1* ^−/−^ mice, where there was preferential selection of iNKT cells at the expense of MAIT cells, possibly by more cDC1s being able to provide more CD1d‐mediated signaling. Thus, positive selection of DP thymocytes in the cortex could be a bottleneck checkpoint required to control the overall numbers of unconventional T cells generated. It is only after positive selection that maturing unconventional (and conventional) αβ T cells move to the medulla [45]. This would also explain the expansion of MAIT cells in the thymus of iNKT‐deficient mice [33].

Overall, our findings have revealed additional layers of the regulation by hematopoietic APCs that contribute to the homeostasis of intrathymic unconventional T cells, at least in C57BL/6 mice.

## 4 Data Limitations and Perspectives

There are several important limitations to our study. First, we did not address the impact of depleting DCs and macrophages on the early CD24^+^ stage in iNKT or MAIT development. CD24^+^ iNKT and MAIT cells are extremely rare cells in the thymus. To accurately quantify these cells will require more rigorous tetramer enrichment strategies than those employed in this study. It is conceivable that hematopoietic antigen‐presenting cells also support these early developmental stages, as well as the effector diversification of mature iNKT and MAIT thymocytes described in our study, and this should be addressed in the future. Second, our study employed the phenomenon of thymic rosettes to demonstrate physical interactions between APC and iNKT/MAIT cells. Evidence of direct interactions in situ are still needed. As the resolution of spatial transcriptomics improves, this could potentially be deployed in the future to map the localization of rare cell types, like iNKT/MAIT cells and APCs, within the thymus. Finally, it is important to acknowledge again that our study focused entirely on the C57BL/6 genetic background, in which iNKT1 and MAIT17 cells are more prominent. As was discussed, the composition of effector iNKT and MAIT subsets can differ between mouse strains and with exposure to different commensal microorganisms, and therefore, APC depletions could also affect the other effector subsets in other contexts.

## 5 Materials and Methods

### 5.1 In Silico Interaction Analyses

Previously published thymic iNKT cell [5], MAIT cell [15], and APC [13] scRNA‐seq datasets from wildtype C57BL/6 mice were analyzed. Cells with <200 RNA counts, >6000 features, or >5% mitochondria genes were filtered out using Seurat (v3.2.2) [46]. The iNKT cell dataset was clustered with the first 12 PCs and visualized at resolution 1.5. The MAIT cell dataset was clustered with the first 10 PCs and visualized at resolution 1. Clusters were assigned the same labels as from their original publications based on marker gene expression. The datasets were then integrated using the RPCA method in Seurat. Interaction analyses were performed using the LIANA package [16]. Differentially expressed genes filtered for surface markers and secreted molecules were extracted (Table S1). Interacting genes were identified using CellphoneDB (v5) and expression scores calculated with the AddFeatureScore function [47].

### 5.2 Animals and Thymus Harvests

Wildtype C57BL/6, *Batf3^−/−^* [20] and *Xcr1* ^−/−^ [48] mice were bred at St Vincent's Institute of Medical Research. *Irf8^lox/lox^ CD11c‐*cre (IRF8ΔDC) mice [10] were obtained from the Walter and Eliza Hall Institute of Medical Research. *Clec9a* ^−/−^ mice [49] were obtained from Monash University. *Mr1* ^−/−^ [50], *Mr1^TdTomato^* [18] and *Mr1^lox/lox^ Lyz2* ^cre^ [18] mice were obtained from the University of Melbourne. Mgl2‐DTR mice [51] were bred and treated with diphtheria toxin (DT) at Malaghan Institute of Medical Research. CX3CR1‐DTR mice [32] were bred and treated with DT at Memorial Sloan Kettering Cancer Center. All facilities were under specific pathogen‐free conditions. All experiments were conducted on age‐ and sex‐matched controls.

DT‐treatment of Mgl2‐DTR and CX3CR1‐DTR mice and their wildtype littermate controls was performed by injecting with 40 ng/g DT in PBS i.p. every 2–3 days for 10 days, commencing at 6–7 weeks of age. Analyses were performed 1 day after the final dose.

For total thymus single cell suspensions, thymuses were minced into small fragments and digested with 0.5 mg/mL Collagenase D and 4 µg/mL DNase I in RPMI + 2% FCS (all from Sigma‐Aldrich), then passed through a mesh. The isolation of thymic rosettes was performed as previously described [13].

### 5.3 FACS

Thymocytes were analyzed either with or without first enriching for iNKT or MAIT cells. iNKT cells were enriched by incubating the single cell suspensions with a CD1d‐PBS44‐PE tetramer, then anti‐PE MACS magnetic beads (Miltenyi). For the enrichment of MAIT cells, the single cell suspensions were first blocked with an MR1‐6FP tetramer before incubating with an MR1‐5‐OP‐RU‐PE tetramer, then with the anti‐PE MACS magnetic beads. Positive selections were then performed on an AutoMACS (Miltenyi).

For cytokine staining, single cell suspensions were restimulated with 50 ng/mL PMA, 1 µg/mL ionomycin, 5 µg/mL brefeldin A, and 2 µM monensin (all from Sigma‐Aldrich) in RPMI + 10% FBS for 3 h prior. For intracellular staining of DCs, the PMA and ionomycin were omitted from the culture.

Staining of cells was performed by first incubating with Fc block (ThermoFisher Scientific) in PBS + 0.5% BSA and 2% FBS for 15 min. If analyzing MAIT cells, the samples were also blocked with an MR1‐6FP tetramer for 15 min. The CD1d‐PBS44 or MR1‐5‐OP‐RU tetramers were then added with the antibody cocktail for staining cell surface proteins for 30 min. DAPI (Sigma‐Aldrich) or Live/Dead fixable blue (ThermoFisher Scientific) was added as a viability stain just before analysis.

For intranuclear staining for transcription factors, the cells were first stained for cell surface markers, then fixed and permeabilized with the Foxp3 transcription factor staining buffer set (ThermoFisher Scientific) before staining for transcription factors.

The conjugated antibodies used are listed in Table S3. Data acquisitions were performed on a BD LSR II (BD Biosciences) or Aurora (Cytek Biosciences) cytometer and analyzed on FlowJo v10.10.0. Sorting of DC and macrophages was performed with a FACS Aria Fusion (BD Biosciences).

### 5.4 In Vitro Culture of Thymic iNKT Cells

Enrichment of thymic iNKT cells was performed as described by Lucas et al. [7] by depleting CD8α^+^ cells. 2 × 10^6^ thymocytes were then plated into 24‐well plates for 72 h in RPMI + 10% FBS (Sigma Aldrich), with or without the addition of sorted APCs or recombinant mouse IL‐12 and IL‐15 (ThermoFisher Scientific).

### 5.5 Stimulation of APCs With 5‐OP‐RU

Single cell suspensions were stimulated with 10 µM of 5‐OP‐RU (or DMSO) in DMEM + 10% FCS for 3 h at 37°C. The cells were washed before staining for MR1 and other cell surface markers for FACS.

### 5.6 QRT‐PCR

Total RNA was extracted from sorted cells with TRIsure (Bioline) and reverse‐transcribed with M‐MuLV reverse transcriptase (NEB). qPCR was performed with GoTaq Green Master Mix (Promega) using the primers *Mr1*‐fwd: TGCTCGCTGTATTCTTGGTG and Mr1‐rev: GAGCTTTCGGCTCCTTCTGT and *Actb*‐fwd: CACAGCTTCTTTGCAGCTCCTT and *Actb*‐rev: CGTCATCCATGGCGAACTG.

### 5.7 Statistical Analysis

Statistical analyses were performed in GraphPad Prism 10 (Version 10.4.1).

## Author Contributions

Y.W., D.G.P., and M.M.W.C. conceived and designed the study. Y.W., T.T.L., S.O., A.R.L., and D.B.M. performed the experiments. Y.W., S.O., A.R.L., A.R.L., D.B.M., G.E.D., F.R., D.G.P., and M.M.W.C. contributed to data analysis. H.E.G.M., T.K., M.H.L., and J.A.V. provided essential materials and scientific discussion. Y.W. and M.M.W.C. wrote the manuscript, and all other authors contributed to review and editing.

## Ethics Statement

Animal experiments were approved by the St Vincent's Hospital Animal Ethics Committee and performed under the Australian code for the care and use of animals for scientific purposes, the Victoria University of Wellington Animal Ethics Committee, or the Institutional Animal Care and Usage Committee at Memorial Sloan Kettering Cancer Center.

## Conflicts of Interest

The authors declare no conflicts of interest.

## Supporting information




**Supporting File**: jssc70490‐sup‐0001‐SuppMat.zip.
