# Supplementary material for: Hematopoietic Antigen Presenting Cells Fine‐Tune the Effector Phenotype of Intrathymic Unconventional αβ T Cells
Source: Eur J Immunol. 2026 Jul 22;56(7):e70243. doi: 10.1002/eji.70243 (PMC13390656; doi:10.1002/eji.70243)
Supplement: Supplementary file 1 — Supporting File: jssc70490‐sup‐0001‐SuppMat.zip. [file EJI-56-e70243-s001.zip › EJI_Supp_Fig.pdf]

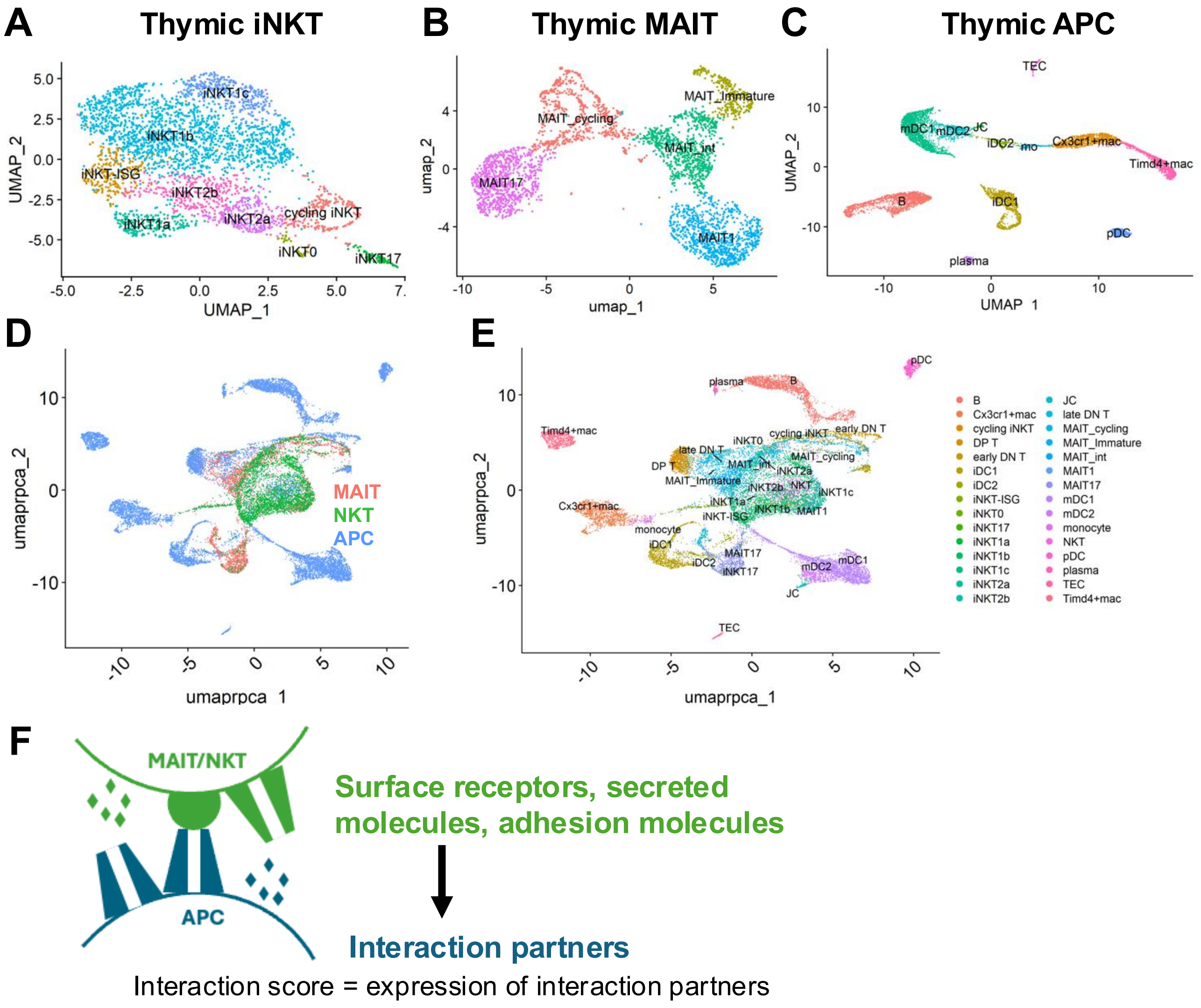

Fig S1. Thymic scRNA-seq datasets used to predict the interaction of APCs with iNKT and MAIT cells. Uniform Manifold Approximation Projection (UMAP) dimensionality reduction plots of scRNA-seq datasets of C57BL/6 mouse thymic **A)** CD1d-PBS57^{+} iNKT cells generated by Baranek, et al (1), **B)** MR1-5-OP-RU^{+}MAIT cells generated Karnaukhov, et al (2), and **C)** hematopoietic APCs that we previously generated (3). Shown are the cell type annotations previously assigned in the original papers. The three datasets were then integrated with the Seurat RPCA method. UMAP plots of the integrated object labelled by **D)** sample of origin or **E)** cell type. **F)** Workflow of how the predicted interaction scores of APC populations with each MAIT/iNKT population were generated. Extracted gene lists are provided in Table S1.

1. T. Baranek *et al.*, High Dimensional Single-Cell Analysis Reveals iNKT Cell Developmental Trajectories and Effector Fate Decision. *Cell Rep* **32**, 108116 (2020).
2. V. K. Karnaukhov *et al.*, Innate-like T cell subset commitment in the murine thymus is independent of TCR characteristics and occurs during proliferation. *Proc Natl Acad Sci U S A* **121**, e2311348121 (2024).
3. Y. Wang *et al.*, Unexpected heterogeneity and tissue-specific properties of the thymic hematopoietic antigen-presenting cell network. *Proc Natl Acad Sci U S A* **122**, e2508184122 (2025).

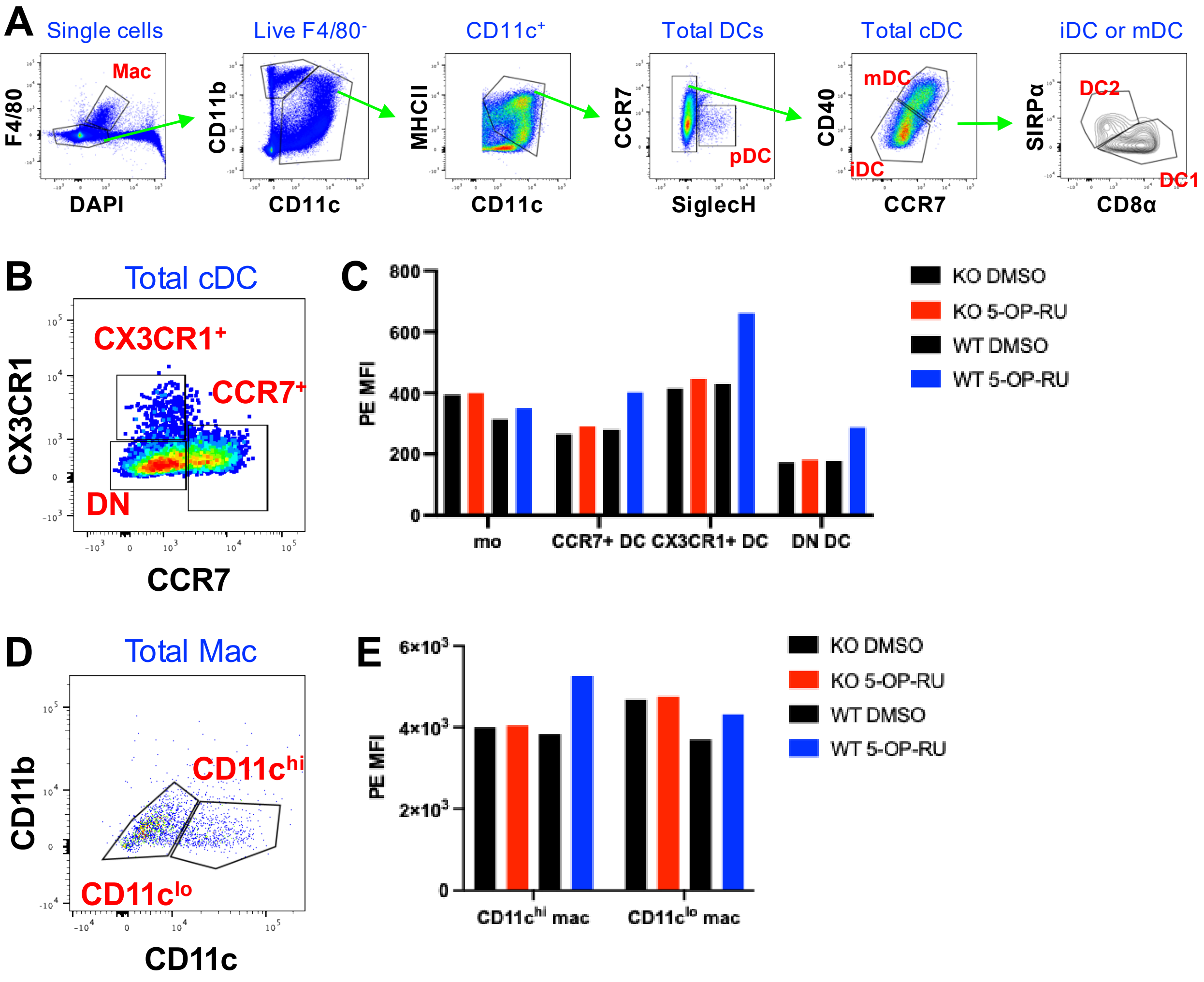

Fig S2. Analysis of thymic myeloid APCs for MR1 expression. A) Gating strategy used to identify myeloid APC populations by FACS. **B)** Representative FACS plot identifying CX3CR1$^+$, CCR7$^+$ and double negative (DN) DCs from the F4/80$^-$CD11c$^+$MHCII$^+$ SiglecH$^-$ cDC gate. **C)** Surface MR1 staining of DCs or monocytes after 3h stimulation with 5-OP-RU or DMSO as a control. Background staining was determined by analyzing *Mr1*$^{-/-}$ (KO) cells **D)** Representative FACS plot identifying CD11c^{lo} and CD11c^{hi} macrophages. CD11c^{lo} macrophages are also TIMD4$^+$, while CD11c^{hi} macrophages are also CX3CR1$^+$ and CD301b$^+$. **E)** Surface MR1 staining of macrophages after 5-OP-RU stimulation.

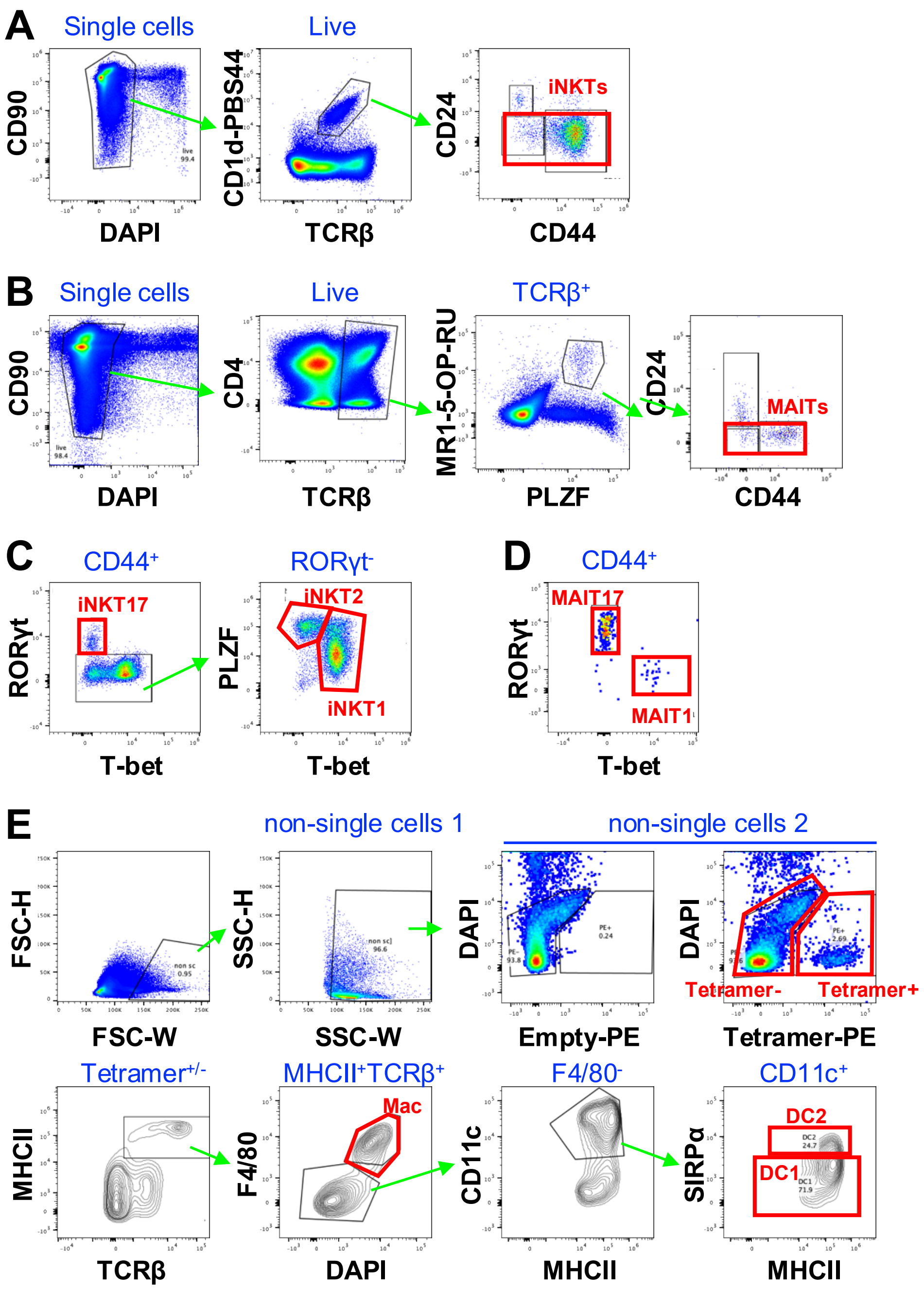

Fig S3. **Gating strategy for identifying iNKT and MAIT cells in the thymus**. **A)** Mature iNKT thymocytes as CD1d-PBS44$^+$TCRβ$^+$CD24$^-$ cells (most of which will also have upregulated CD44). **B)** Mature MAIT thymocytes are identified as MR1-5-OP-RU$^+$TCRβ$^+$PLZF$^+$CD24$^-$ cells (most of which will also have upregulated CD44). Prior to staining with the MR1-5-OP-RU tetramer, the cells were first blocked with MR1-6FP. **C)** Subdividing mature iNKT subsets intranuclear staining for RORγt, PLZF and T-bet. **D)** Subdividing mature MAIT subsets by intranuclear staining for RORγt and T-bet. **E)** Assessing interaction of iNKT/MAIT cells with APCs within thymic rosettes by FACS. Whole rosettes were identified as "large cells" by forward and side scatter, then gating on tetramer$^+$ or tetramer$^-$ for assessment of APC markers.

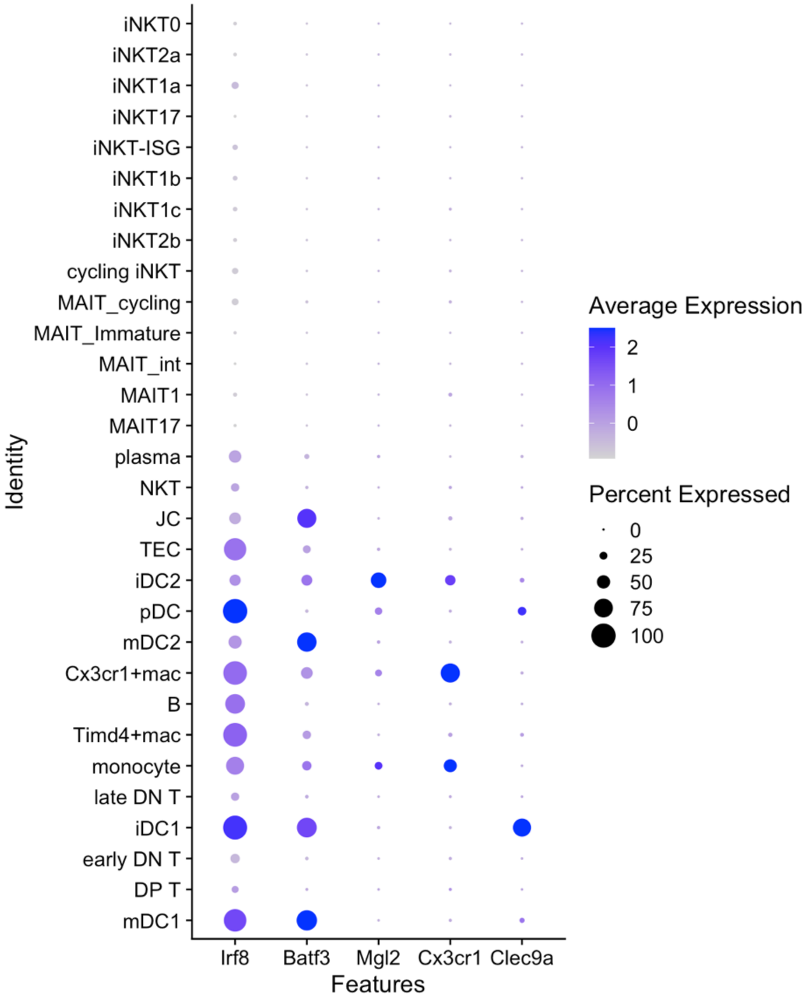

Fig S4. Expression profiles of the genes targeted for deletion or used to drive DTR expression. The populations shown correspond to the clusters identified in the scRNAseq datasets in Fig S1. Although *Irf8* has relatively broad expression, conditional gene inactivation with CD11c-cre was employed to achieve DC1 deficiency.

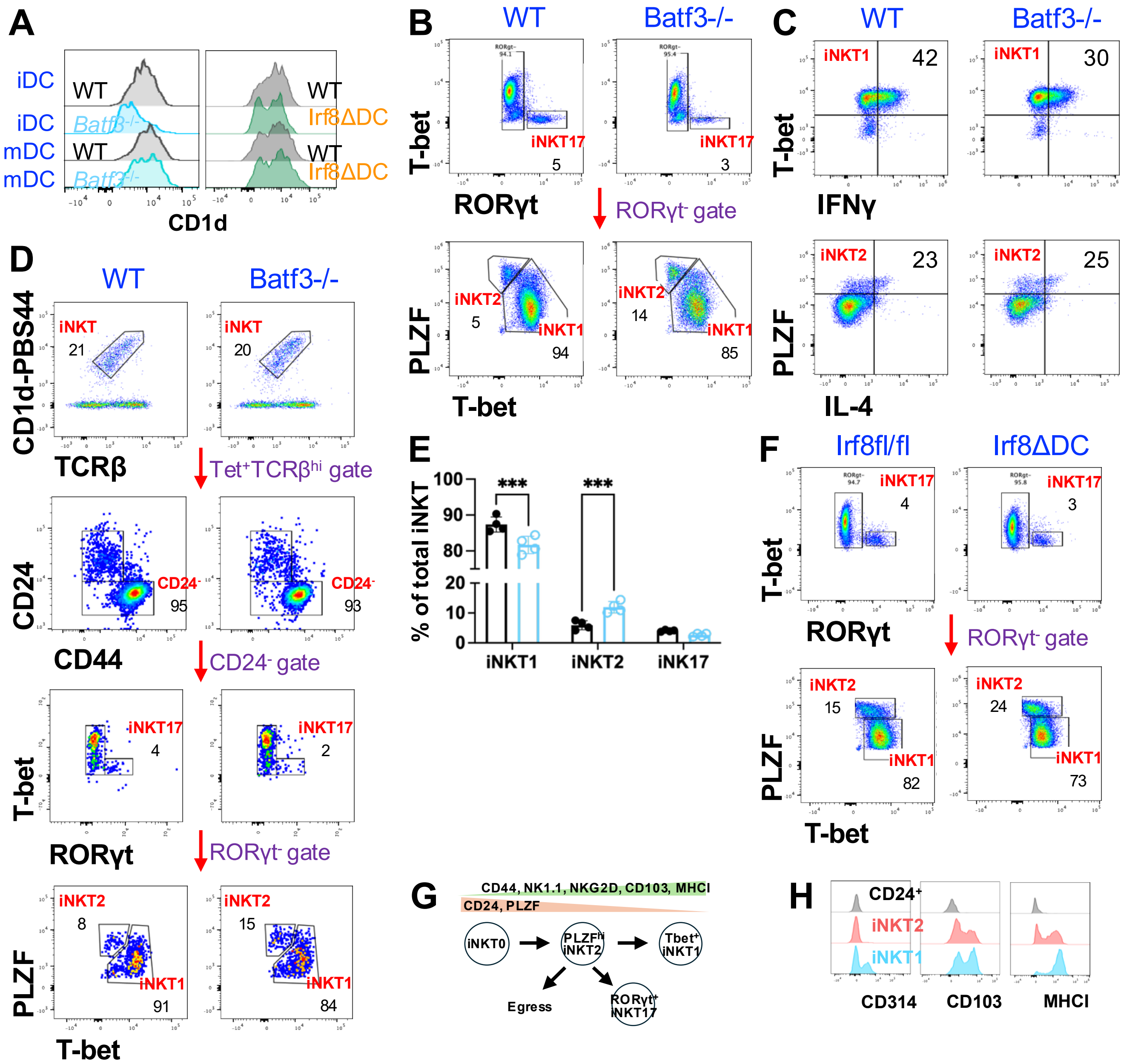

Fig S5. Additional analyses of thymic iNKT cells in DC1 deficient mice. A) Representative FACS analysis for surface CD1d expression on immature (CD86^{lo}) and mature (CD86^{hi}) DCs in *Batf3*$^{-/-}$ and Irf8ΔDC mice. **B)** Representative FACS comparing subsets within total mature iNKT thymocytes in *Batf3*$^{-/-}$ mice based on transcription factor expression. **C)** Representative intracellular cytokine FACS analysis in *Batf3*$^{-/-}$ mice following restimulation with PMA/ionomycin for 3h. Shown is the staining gated on mature iNKT cells. The numbers indicate the percentage of iNKT1 cells that express IFNγ (upper panels) or iNKT2 cells that express IL-4 (lower panels). **D/E)** Analysis of thymic iNKT cells in *Batf3*$^{-/-}$ mice after labelling with CD1d-PBS44-PE and enrichment with anti-PE MACS magnetic beads. Shown is the FACS analysis of a representative pair of mice (D) and the pooled data (E). Each dot is of an individual animal analyzed (n=4), where each experiment involved the analysis of a *Batf3*$^{-/-}$ and WT littermate pair.. *** *P*<0.005 (two-way ANOVA with Šídák's multiple comparisons test). **F)** Representative FACS comparing subsets within total mature iNKT cells in Irf8ΔDC mice based on transcription factor expression. **G)** Developmental trajectory of thymic iNKT cells summarizing changes in marker expression. **H)** Representative FACS of iNKT subsets for marker expression.

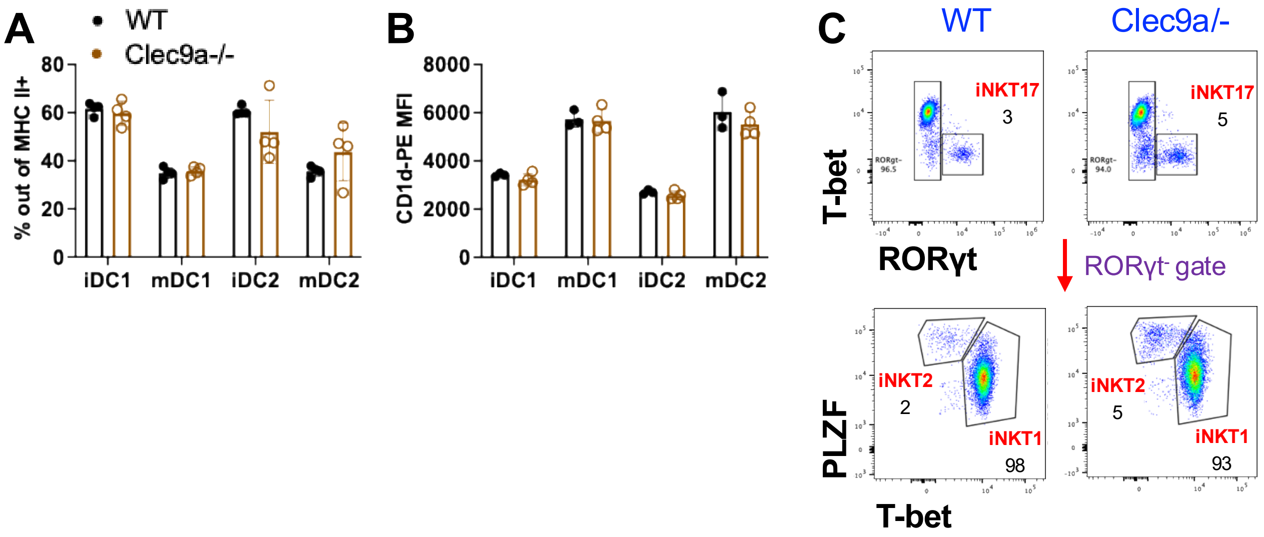

Fig S6. Additional analyses of CLEC9A deficient mice. A) The thymus *Clec9a*$^{-/-}$ mice were analyzed for the frequency of CD86^{-} immature and CD86^{+} mature DC subsets by FACS. Shown are the frequencies out of total MHCII^{+} APCs. **B)** FACS analysis for surface CD1d expression by the indicated DC subsets. **C)** Representative FACS comparing subsets within CD24^{-} iNKT cells in *Clec9a*$^{-/-}$ mice based on transcription factor expression. For column graphs, each dot is of an individual animal analyzed (n=4), where each experiment involved the analysis of a *Clec9a*$^{-/-}$ and WT littermate pair.

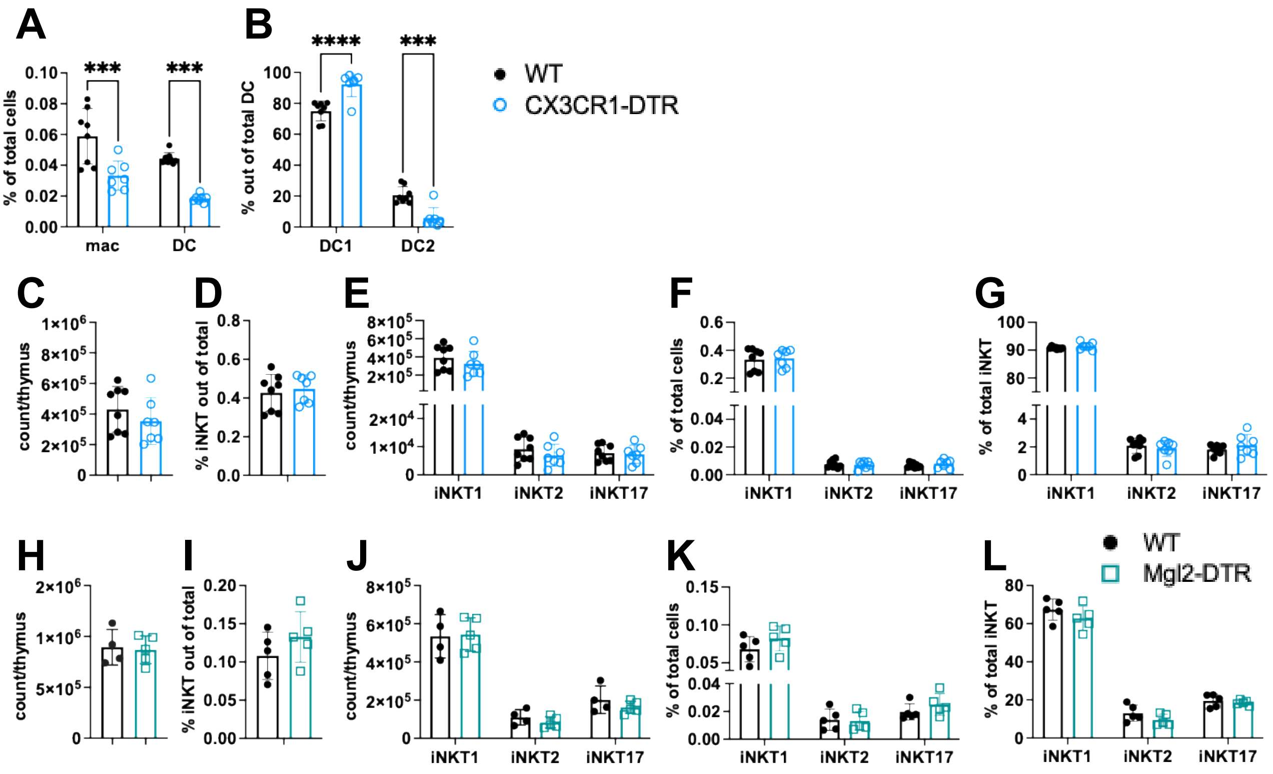

Fig S7. Thymic iNKT cells are unaffected in DC2/macrophage-depleted mice. A) CX3CR1-DTR mice and WT littermates (n=8) were DT-treated for 10d, then the thymus was analyzed by FACS for the depletion of macrophages and DCs. **B)** DCs were subdivided into DC1 versus DC2 based on SIRPα and CD8α expression. The thymus of DT-treated CX3CR1-DTR or littermate WT mice (n=8) were analyzed by FACS for **C)** the number of mature iNKT cells per thymus, **D)** % iNKT out of total thymocytes, **E)** the number of each iNKT effector subset per thymus, **F)** iNKT effector subset out of total thymocytes, and **G)** effector subset out of total iNKT cells. The thymus of DT-treated Mgl2-DTR or littermate mice (n=5) were analyzed for **H)** the number of mature iNKT cells per thymus, **I)** % iNKT out of total thymocytes, **J)** the number of each iNKT effector subset per thymus, **K)** iNKT effector subset out of total thymocytes, and **L)** effector subset out of total iNKT cells. For all column graphs, each dot is of an individual animal analyzed, For column graphs, each dot is of an individual animal analyzed, where each experiment involved the analysis of a DTR and WT littermate pair. *** P<0.005, **** P<0.001 (Two-way ANOVA with Šídák's multiple comparisons test).

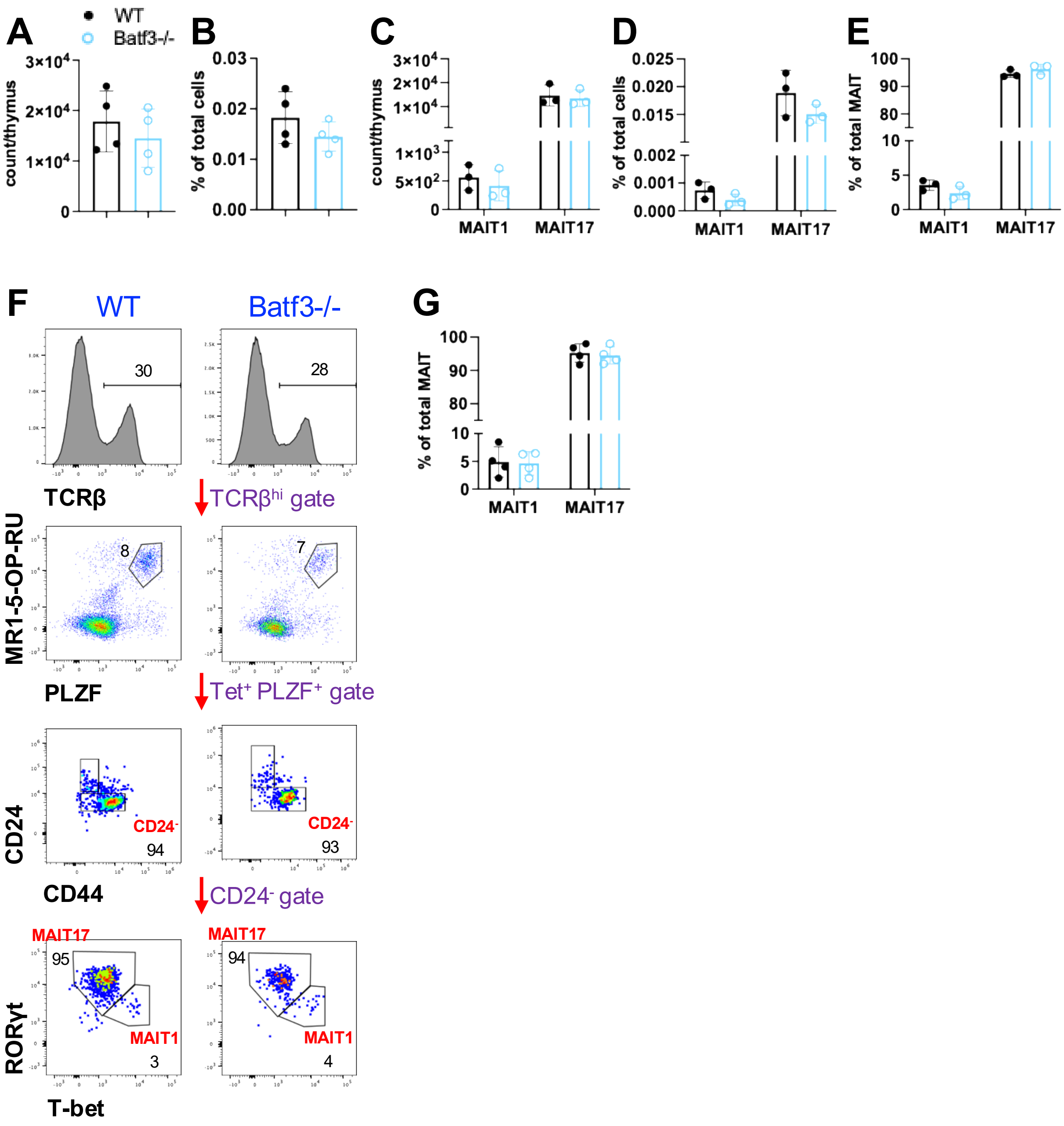

Fig S8. Analysis of thymic MAIT cells in BATF3-deficient mice. FACS analysis of *Batf3*$^{-/-}$ mice for **A)** the number of mature MAIT cells per thymus (n=4), **B)** % MAIT out of total thymocytes, **C)** number of MAIT effector subset per thymus (n=3), **D)** effector subset out of total thymocytes, and **E)** effector subset out of total MAIT cells. **F/G)** Analysis of thymic MAIT cells in *Batf3*$^{-/-}$ mice after blocking with MR1-6FP, labelling with MR1-5-OP-RU-PE and enrichment with anti-PE MACS magnetic beads. Shown is **F)** the FACS analysis of a representative pair of mice and **G)** the pooled data (n=4) for effector subsets out of total MAIT cells. For column graphs, each dot is of an individual animal analyzed, where each experiment involved the analysis of a *Batf3*$^{-/-}$ and WT littermate pair. No statistical difference found (Two-way ANOVA with Šídák's multiple comparisons test or unpaired T-test).

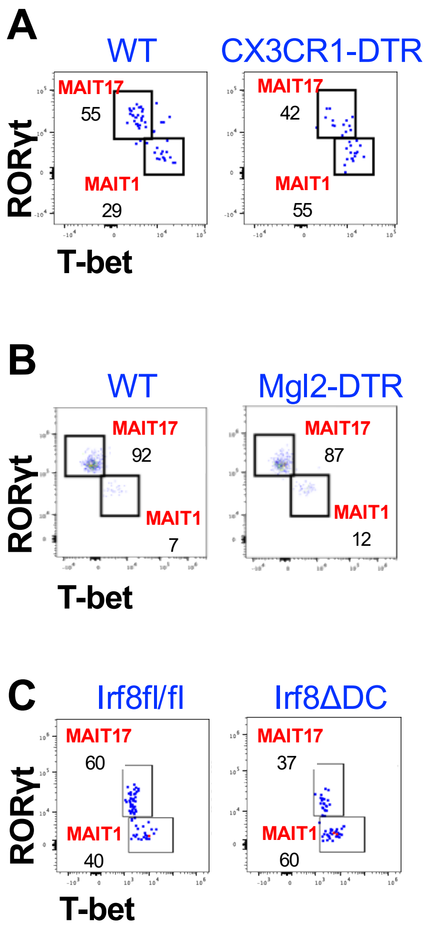

Fig S9. Additional analyses of thymic MAIT cells in macrophage and DC2-depleted mice. A) CX3CR1-DTR mice and WT littermates were DT-treated for 10d, then the thymus was analyzed for mature MAIT cells by FACS. Shown is a representative FACS for effector subset out of total MAIT cells. **B)** Representative FACS for effectors subset out of total MAIT cells in the thymus of DT-treated Mgl2-DTR mice. **C)** Representative FACS for effectors subset out of total MAIT cells in the thymus of Irf8ΔDC mice.

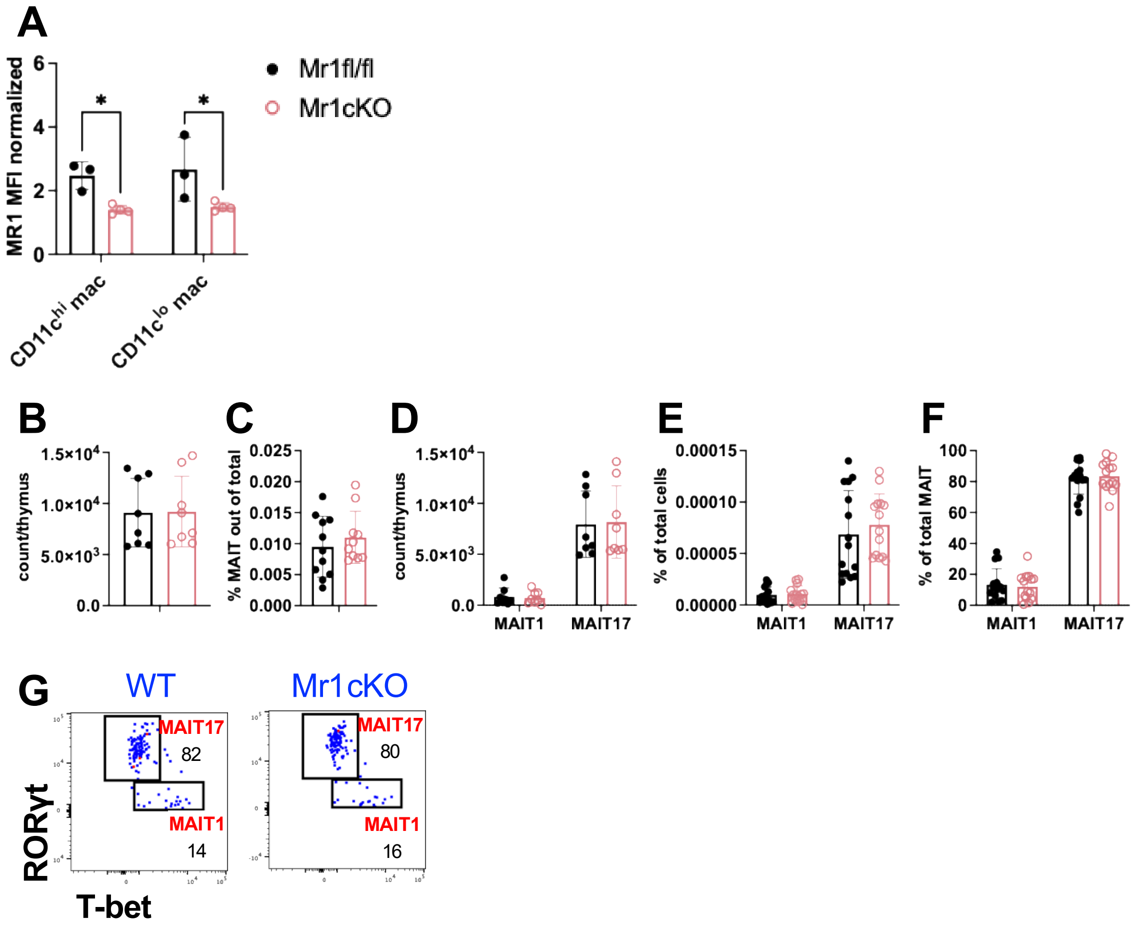

Fig S10. Analyses macrophage-specific MR1 deficient mice. A) FACS analysis of steady state surface MR1 on macrophage subsets in the thymus of *Mr1^fl/fl^ Lyz2^cre^* conditional knockout (MR1cKO) mice (n=3). Expression was normalized to cells stained with secondary antibody and without the anti-MR1 primary antibody. MR1cKO and littermate control mice were analyzed by FACS for **B)** the number of mature MAIT cells per thymus, **C)** % MAIT out of total thymocytes, **D)** the number of each MAIT effector subset per thymus, **F)** MAIT effector subset out of total thymocytes, and **G)** effector subset out of total MAIT cells. **G)** Representative FACS comparing subsets within MAIT cells based on transcription factor expression. For column graphs, each dot is of an individual animal analyzed, where each experiment involved the analysis of a MR1cKO and *Mr1^fl/fl^* littermate pair. * P<0.05 (Two-way ANOVA with Šídák's multiple comparisons test or unpaired T-test).

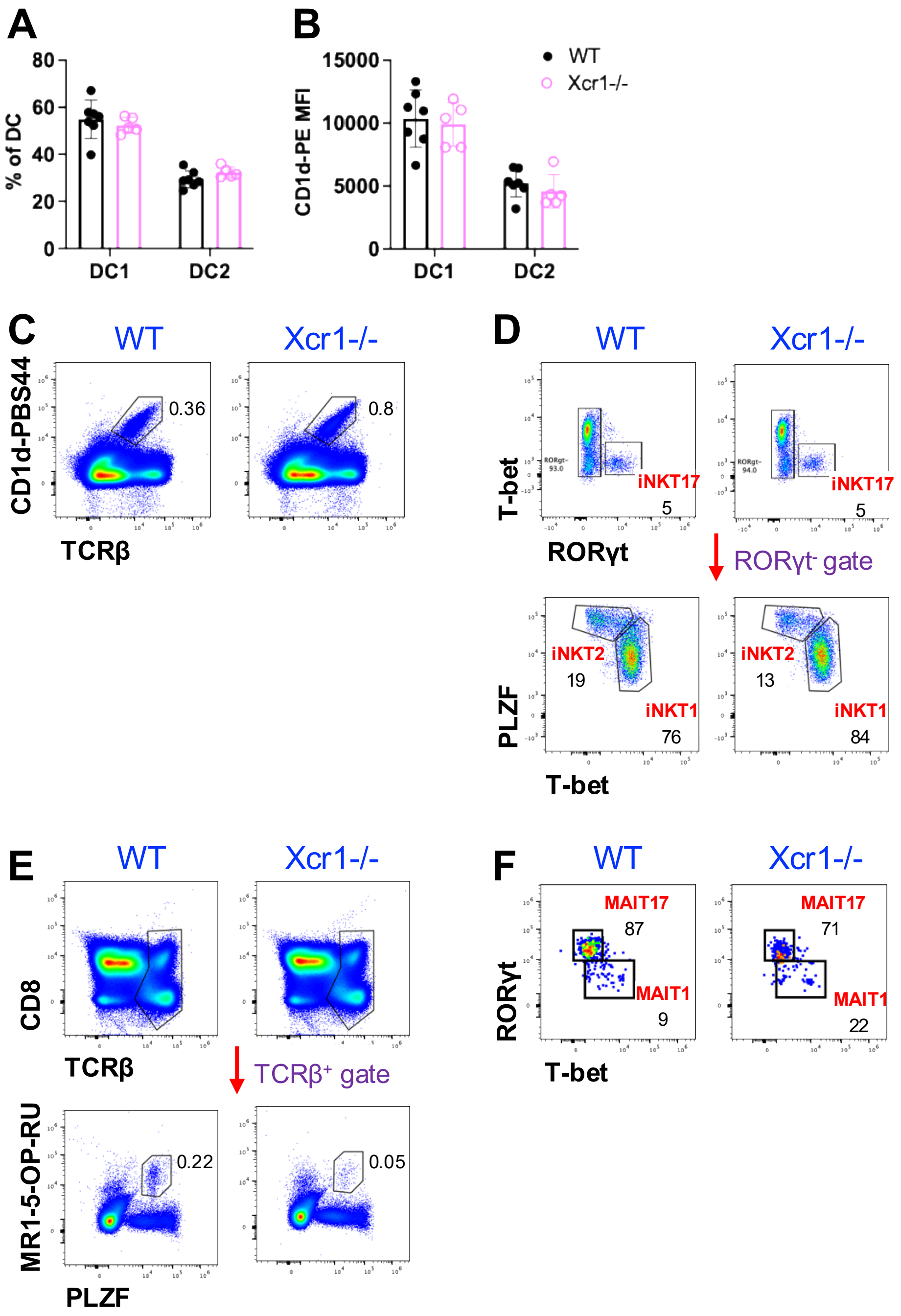

Fig S11. Additional analyses of thymic MAIT and iNKT cells in XCR1 deficient mice. Analysis of **A)** DC subsets and **B)** surface CD1d expression by FACS. **C)** Representative FACS for total CD1d-PBS44$^+$TCRβ$^+$ iNKT cells in the thymus. **D)** Representative FACS comparing subsets within mature iNKT cells based on transcription factor expression. **E)** Representative FACS for total MR1-5-OP-RU$^+$TCRβ$^+$PLZF$^+$ MAIT cells in the thymus **F)** Representative FACS comparing subsets within mature MAIT cells based on transcription factor expression. For column graphs, each dot is of an individual animal analyzed (n=5-7), where each experiment involved the analysis of a *Xcr1*$^{-/-}$ and 1 or 2 WT littermate pair. Statistical testing: two-way ANOVA with Šídák's multiple comparisons test (not difference found).

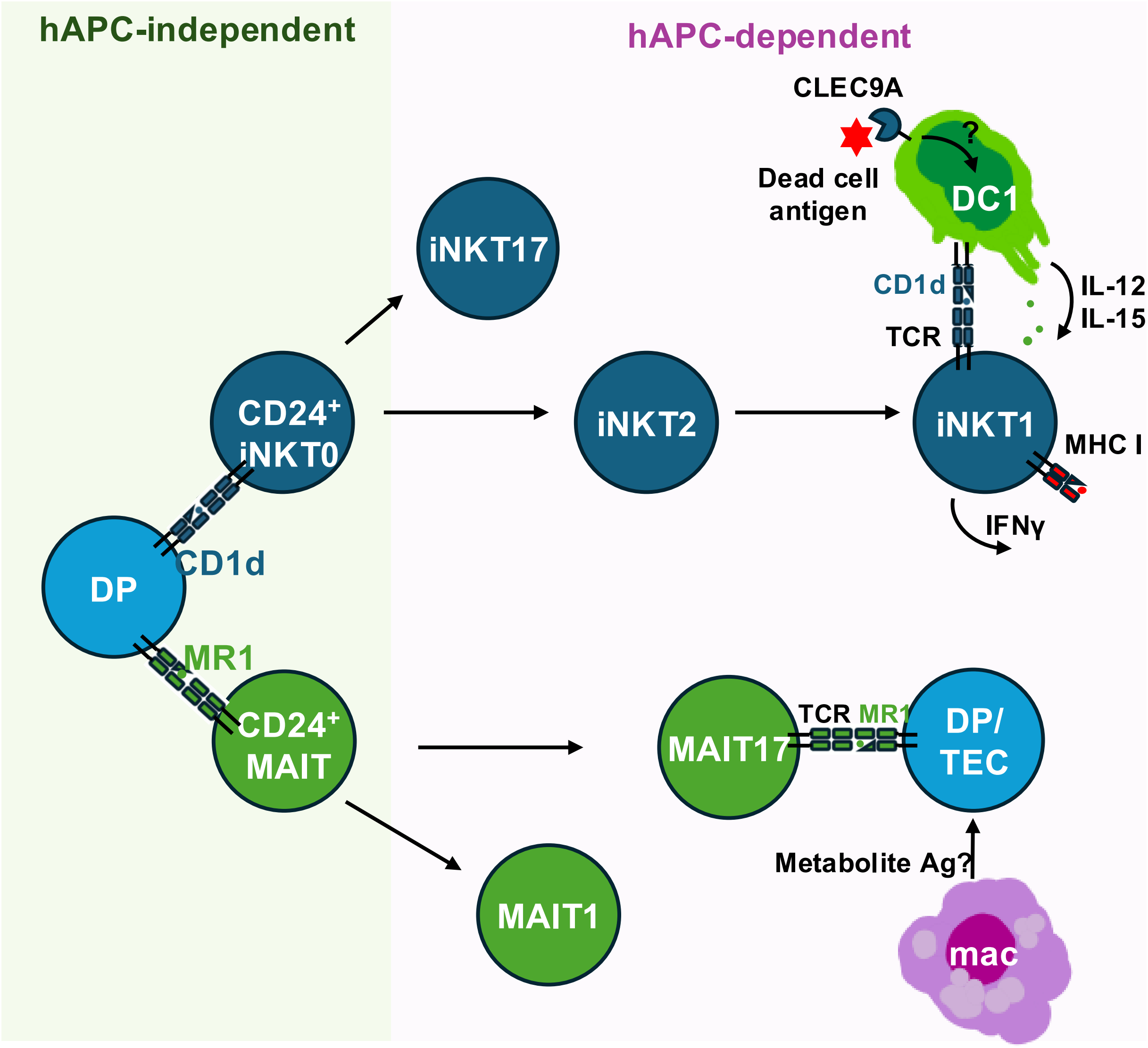

Fig S12. Proposed model of how hematopoietic APCs fine-tune the differentiation and maintenance of effector unconventional αβ T cells in the thymus. At the earliest stages, the positive selection of immature iNKT and MAIT cells compete for the same niche (i.e. DP thymocytes). Following migration to the medulla, maturation of effector iNKT and MAIT cells are then dependent on guiding signals from distinct hAPCs: DC1s for iNKT cells and macrophages for MAIT cells.
